# A high-throughput genetic screen identifies previously uncharacterized *Borrelia burgdorferi* genes important for resistance against reactive oxygen and nitrogen species

**DOI:** 10.1371/journal.ppat.1006225

**Published:** 2017-02-17

**Authors:** Meghan E. Ramsey, Jenny A. Hyde, Diana N. Medina-Perez, Tao Lin, Lihui Gao, Maureen E. Lundt, Xin Li, Steven J. Norris, Jon T. Skare, Linden T. Hu

**Affiliations:** 1 Department of Molecular Biology and Microbiology, Tufts University, Boston, Massachusetts, United States of America; 2 Department of Microbial Pathogenesis and Immunology, College of Medicine, Texas A&M Health Sciences Center, Bryan, Texas, United States of America; 3 Department of Pathology and Laboratory Medicine, McGovern Medical School at UTHealth, Houston, Texas, United States of America; 4 Division of Geographic Medicine and Infectious Diseases, Tufts Medical Center, Boston, Massachusetts, United States of America; Stanford University School of Medicine, UNITED STATES

## Abstract

*Borrelia burgdorferi*, the causative agent of Lyme disease in humans, is exposed to reactive oxygen and nitrogen species (ROS and RNS) in both the tick vector and vertebrate reservoir hosts. *B*. *burgdorferi* contains a limited repertoire of canonical oxidative stress response genes, suggesting that novel gene functions may be important for protection of *B*. *burgdorferi* against ROS or RNS exposure. Here, we use transposon insertion sequencing (Tn-seq) to conduct an unbiased search for genes involved in resistance to nitric oxide, hydrogen peroxide, and tertiary-butyl hydroperoxide *in vitro*. The screens identified 66 genes whose disruption resulted in increased susceptibility to at least one of the stressors. These genes include previously characterized mediators of ROS and RNS resistance (including components of the nucleotide excision repair pathway and a subunit of a riboflavin transporter), as well as novel putative resistance candidates. DNA repair mutants were among the most sensitive to RNS in the Tn-seq screen, and survival assays with individual Tn mutants confirmed that the putative ribonuclease BB0839 is involved in resistance to nitric oxide. In contrast, mutants lacking predicted inner membrane proteins or transporters were among the most sensitive to ROS, and the contribution of three such membrane proteins (BB0017, BB0164, and BB0202) to ROS sensitivity was confirmed using individual Tn mutants and complemented strains. Further analysis showed that levels of intracellular manganese are significantly reduced in the Tn::*bb0164* mutant, identifying a novel role for BB0164 in *B*. *burgdorferi* manganese homeostasis. Infection of C57BL/6 and *gp91*^*phox*-/-^ mice with a mini-library of 39 Tn mutants showed that many of the genes identified in the *in vitro* screens are required for infectivity in mice. Collectively, our data provide insight into how *B*. *burgdorferi* responds to ROS and RNS and suggests that this response is relevant to the *in vivo* success of the organism.

## Author summary

*Borrelia burgdorferi* is the causative agent of Lyme disease, the most common vector-borne disease in the United States. *B*. *burgdorferi* is maintained in the environment in a complex cycle in which it is transmitted between a tick vector and small vertebrate reservoir hosts. Because of this, *B*. *burgdorferi* must be able to adapt to changing environmental conditions. Historically, this organism has been difficult to manipulate genetically in the lab. We have taken advantage of recent technological advances to conduct a global search for genes that enable the spirochete to defend itself against reactive oxygen and nitrogen species produced by the host immune system. Using transposon insertion sequencing (Tn-seq), we exposed a pool of *B*. *burgdorferi* mutants to reactive oxygen or nitrogen species and used high-throughput sequencing to quantify the frequency of each individual mutant before and after the stress exposure. We found that genes encoding DNA repair enzymes and predicted inner membrane proteins are important for resistance to RNS and ROS, respectively. Understanding how *B*. *burgdorferi* responds to the stresses and changing environmental conditions it experiences as it transitions between its tick vector and vertebrate hosts potentially will identify new targets for interrupting its life cycle.

## Introduction

The spirochete *Borrelia burgdorferi* is the causative agent of Lyme disease and the most common arthropod-borne disease in the United States. The spirochete is maintained in the environment within a complex enzootic cycle involving *Ixodes* ticks and a diversity of mammalian and avian reservoirs [1]. As a result, *B*. *burgdorferi* must adapt to changing host environments. A number of studies have chronicled how changes in environmental factors such as temperature, pH, cell density, metals, and dissolved oxygen and carbon dioxide levels affect gene expression in this organism [2–8]. Reactive oxygen and nitrogen species (ROS and RNS) represent an additional and less well-studied component of the host environment to which *B*. *burgdorferi* must sense and respond. ROS and RNS are detectable in the salivary glands and midgut of *Ixodes* ticks following a bloodmeal [9] and are also likely present in the vertebrate host at the tick bite site due to the recruitment of inflammatory cells to this site [10, 11].

ROS and RNS are toxic to *B*. *burgdorferi*, although the targets of oxidative damage are different in this organism compared to well-studied model organisms like *Escherichia coli* [12–14]. In *E*. *coli*, DNA is a primary target of oxidative damage because of the Fenton reaction, in which hydrogen peroxide (H_2_O_2_) reacts with cellular iron to form the highly reactive hydroxyl radical [15, 16]. In contrast to *E*. *coli*, DNA damage is not detectable in *B*. *burgdorferi* after exposure to H_2_O_2_ or nitric oxide (NO) [12, 13]. Subsequent studies suggested that the apparent lack of DNA damage is due to efficient repair mechanisms: DNA damage is evident in *B*. *burgdorferi* mutants lacking components of the nucleotide excision repair (NER) pathway after NO exposure, while DNA repair mutants lacking UvrA, UvrB and MutS show decreased survival relative to WT bacteria after H_2_O_2_ exposure [12, 14, 17]. Differences in DNA damage between *B*. *burgdorferi* and *E*. *coli* may also be due to differences in iron utilization. *B*. *burgdorferi* can grow in iron-depleted media and does not appear to import iron into the cell [18]. However, a recent study found appreciable intracellular levels of iron in *B*. *burgdorferi* and identified a multifunctional ferritin-like protein that binds both copper and iron [19]. It remains unclear what biological role this iron may be playing in *B*. *burgdorferi* since no iron-requiring enzymes have been characterized to date [20]. Instead of DNA damage, lipid peroxidation may play an important role in ROS toxicity in *Borrelia* organisms [13]. *B*. *burgdorferi* does not synthesize its own lipids and instead scavenges them from its eukaryotic hosts. As a result, polyunsaturated lipids are present in its membranes and are susceptible to peroxidation [13]. Nitrosylation of cysteine thiols, notably those of zinc metalloproteins, is a primary effect of NO in the cell and thus may be important in the toxicity of this compound in *B*. *burgdorferi* [12].

*B*. *burgdorferi* also appears to encode a more limited repertoire of ROS and RNS detoxifying proteins compared to *E*. *coli*. Genes known to be involved in ROS resistance in *B*. *burgdorferi* include a manganese-dependent superoxide dismutase (SodA), a manganese transporter (BmtA), and a riboflavin ATP-binding cassette transport system [21–23]. BicA (also called Dps or NapA) is involved in metal homeostasis, and *bicA* mutants are actually more resistant to oxidative stress under metal replete conditions [19]. The genome also encodes homologs of sulfoxide reductase, thioredoxin, and thioredoxin reductase, none of which have been functionally characterized. The genome does not encode any peroxidases or catalases. Instead, coenzyme A reductase (Cdr) has been implicated in both H_2_O_2_ detoxification and thiol/disulphide redox control [24, 25]. Since the genome does not encode glutathione reductase and glutathione is not detectable in the cell, coenzyme A is thought to serve as the primary low molecular weight thiol in *B*. *burgdorferi* [24]. Also lacking from the *B*. *burgdorferi* genome are homologs of canonical oxidative stress response regulators like SoxRS or OxyR [20]. The Fur/Per homolog BosR has been shown to regulate genes important for oxidative stress resistance, metal homeostasis, and virulence, but its role in the *B*. *burgdorferi* oxidative stress response is not well characterized [26–29].

Given the biochemical differences between *B*. *burgdorferi* and well-studied model organisms, as well as the lack of canonical detoxification enzymes, we undertook an unbiased search for additional factors involved in resistance against ROS and RNS. We exposed a previously described *B*. *burgdorferi* transposon (Tn) library to ROS and RNS [30]. We then used transposon insertion sequencing (Tn-seq) to identify Tn mutants that decreased in frequency after stress exposure [31, 32]. Based on the results of the *in vitro* screens, which identified both previously characterized ROS and RNS resistance genes as well as novel resistance candidates, we generated a mini-library of Tn mutants and used Tn-seq to assess the ability of these mutants to infect both wild-type and superoxide-deficient mice. The majority of these mutants exhibited reduced fitness *in vivo*. Overall, these studies provide insight into the *B*. *burgdorferi* response to ROS and RNS and serve as a first step to begin characterizing its novel approach to oxidative detoxification.

## Results

### Optimization of experimental conditions for an *in vitro* Tn-seq screen

**Fig 1:**
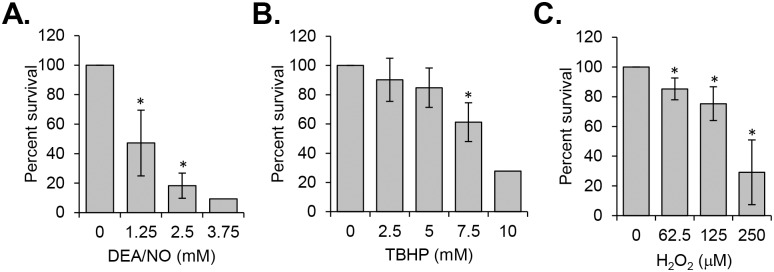
Susceptibility of *B*. *burgdorferi* strain B31 5A18NP1 to ROS and RNS. The percent survival of *B*. *burgdorferi* B31 5A18NP1, the parental strain of the Tn library, was determined following exposure to DEA/NO for two hours in BSK-II (A) or to TBHP (B) or H_2_O_2_ (C) for four hours in Modified BSK-II. Bacteria were exposed to increasing concentrations of ROS or RNS. Percent survival was determined by plating bacteria in semi-solid agarose overlays and comparing colony forming units to an untreated control. The average ± standard deviation of three independent experiments is shown in most cases. Data points without error bars represent the median of two independent experiments. *, significantly different from the untreated control (*P* < 0.05) by Student’s two-tailed *t*-test.

The arrayed *B*. *burgdorferi* Tn library was constructed in the infectious, transformable B31 5A18NP1 strain background and contains 6,625 Tn mutants [30]. We decided to screen the library for sensitivity against two ROS molecules, hydrogen peroxide (H_2_O_2_) and the alkyl peroxide tertiary-butyl hydroperoxide (TBHP), as well as the NO-donor diethylamine NONOate (DEA/NO). NO and H_2_O_2_ were selected since they are molecules produced by innate immune cells *in vivo* [33], while TBHP was selected because it induces lipid oxidation in *B*. *burgdorferi* [13]. We began by determining the susceptibility of 5A18NP1 to TBHP, H_2_O_2_, and DEA/NO (Fig 1). Because pyruvate in the complex *Borrelia* growth medium provides passive protection against H_2_O_2_ [14], ROS exposures were conducted in a modified medium (Modified BSK-II) lacking pyruvate, bovine serum albumin, and rabbit serum. TBHP and DEA/NO caused killing of 5A18NP1 at millimolar concentrations (Fig 1A and 1B). 5A18NP1 was more sensitive to H_2_O_2_, with significant killing occurring at micromolar concentrations (Fig 1C).

The ideal conditions for the Tn-seq screen would result in the death of Tn mutants with insertions in genes involved in ROS and RNS resistance, but would allow survival of Tn mutants with insertions in “neutral” genes. To identify these conditions, we conducted a pilot Tn-seq study using DEA/NO as the stressor. Although the Tn library does not contain mutants with insertions in many of the known *B*. *burgdorferi* ROS and RNS resistance genes, it does contain mutants with insertions in *uvrB*, *uvrC*, *and uvrD*, all of which encode components of the NER pathway that are required for repairing DNA damage after NO exposure [12, 34]. These mutants were used as internal controls. Since Tn-seq relies on the detection of genomic DNA to quantify the frequency of Tn mutants, an outgrowth step was added after the stress exposure to dilute out any DNA originating from bacteria that had died. Exposure of the library to a low concentration of DEA/NO (1.25 mM, corresponding to approximately 50% survival of the parental strain, Fig 1A) resulted in only modest decreases in the frequency of NER pathway mutants compared to the untreated library, in some cases less than two-fold. However, after exposure to a higher concentration (2.5 mM, corresponding to approximately 20% survival of the parental strain, Fig 1A), Tn mutants with insertions in *uvrB*, *uvrC*, or *uvrD* all decreased more than 10-fold in frequency compared to the untreated library (S1 and S2 Tables). Under these same conditions, the majority of Tn mutants in the library changed less than 2-fold in frequency, consistent with the prediction that the majority of the genes in the genome are not involved in NO resistance. Based on the results of the pilot DEA/NO screen, we chose to conduct the ROS screens using 0.25 mM H_2_O_2_ and 10 mM TBHP, concentrations that corresponded to similar levels of toxicity for the parental strain (Fig 1).

### *In vitro* Tn-seq screens identify genes involved in NO, H_2_O_2_, or TBHP resistance

Two independent cultures of the *B*. *burgdorferi* Tn library were exposed to 2.5 mM DEA/NO, 10 mM TBHP, 0.25 mM H_2_O_2_, or culture medium alone. The frequency of individual Tn mutants in the treated and untreated samples was determined using Tn-seq. Reproducibility was high between the two replicates in each case, with Spearman correlation coefficients R > 0.93 (S1 Fig). A frequency ratio was then calculated for each Tn mutant in the library by comparing its frequency in the treated library to its frequency in the untreated library (S1 Table). We required each Tn mutant to be represented by at least 10 sequence reads in both replicates of the untreated libraries (out of a total of approximately 5×10^6^ sequence reads) to be included in this analysis. A frequency ratio less than one indicates that the Tn mutant decreased in frequency after stress exposure, suggesting that the disrupted gene is involved in ROS or RNS resistance. On the other hand, a frequency ratio greater than one indicates that the Tn mutant increased in frequency after stress exposure, suggesting that the disrupted gene actually sensitizes the cell to ROS or RNS. An overall frequency ratio was also calculated at the gene level by aggregating all of the sequence reads mapping within the same gene (S2 Table). These two analyses, one at the level of the individual Tn mutant and one at the level of the gene, provide slightly different information, and we took both into account when analyzing the data and deciding which genes to investigate further. In order to prioritize genes for subsequent studies, we considered frequency ratios between 0.5 and 2 (i.e. less than a 2-fold change in frequency) to be neutral.

**Fig 2:**
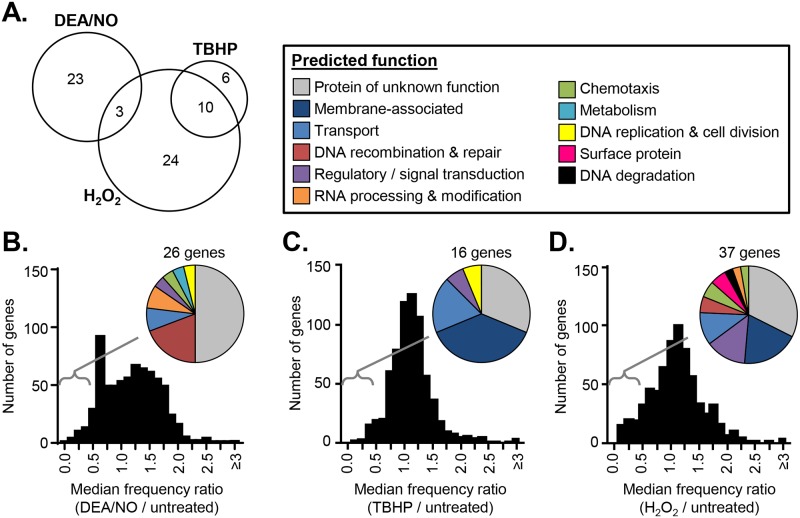
Summary of *in vitro* Tn-seq screen results. An overall frequency ratio was determined for each gene by comparing the frequency of sequence reads mapping within the gene in the treated library compared to the untreated library. (A) The number of genes with overall frequency ratios <0.5 in both replicates of the DEA/NO, TBHP, or H_2_O_2_ Tn-seq screens. The distribution of the median overall frequency ratios at the gene level is shown after exposure to 2.5 mM DEA/NO (B), 10 mM TBHP (C), or 0.25 mM H_2_O_2_ (D). Pie charts indicate the predicted functions of the genes with overall frequency ratios <0.5 in each case.

In all three screens, the majority of genes had neutral overall frequency ratios (Fig 2). We identified 66 genes that had overall frequency ratios below our neutral fitness cut-off value of 0.5 (Fig 2A, S3 Table). Of the three screens, we identified the most genes in the H_2_O_2_ screen (37 genes, Fig 2A, S3 Table). Twenty-six genes were identified in the DEA/NO screen, and three of these genes overlapped with those identified in the H_2_O_2_ screen (Fig 2, S3 Table). We identified the fewest genes in the TBHP screen (only 16), but most of them were also identified in the H_2_O_2_ screen (Fig 2, S3 Table). Many of the genes identified in the three screens have no predicted function and have not been previously characterized (Fig 2, S3 Table). Approximately 20% of the genes that were identified in the DEA/NO screen encode DNA repair enzymes, consistent with previous reports that DNA repair enzymes are involved in NO resistance in *B*. *burgdorferi* (Fig 2B) [12, 34]. In contrast, approximately 40% of the genes identified in the TBHP and H_2_O_2_ screens encode predicted inner membrane proteins or transporters (Fig 2C and 2D). Twenty-one genes were identified that had overall frequency ratios above 2, with most of these genes being identified in the H_2_O_2_ or TBHP screens (S4 Table).

### Validation of a putative NO resistance gene

The DEA/NO screen identified nine genes with overall frequency ratios less than 0.33 in both replicates (Table 1). The genes *uvrB*, *uvrC*, and *uvrD* had some of the lowest overall frequency ratios in the screen (median ratios of 0.072, 0.048, and 0.103, respectively), consistent with their known role in NO resistance [12, 34] (Table 1). A gene encoding a putative ribonuclease (*bb0839*) also had a low overall frequency ratio (median ratio of 0.074, Table 1). The remaining five genes with frequency ratios below 0.33 encode hypothetical proteins (*bba54*, *bb0617*, and *bb0267*), a putative laccase domain-containing protein (*bb0467*), and a CobQ/CobB/MinD/ParA nucleotide binding domain containing protein (*bb0431*) (Table 1) [20, 35].

**Table 1:** Putative *B*. *burgdorferi* genes involved in RNS and ROS resistance.

Stress condition	Replicon	Locus	Annotation / Predicted function	Median Frequency Ratio (Replicate 1, Replicate 2)
DEA/NO	chr	*bb0457*	UvrC, excinuclease ABC subunit	0.048 (0.054, 0.041)
	chr	*bb0836*	UvrB, excinuclease ABC subunit	0.072 (0.110, 0.034)
	chr	*bb0839*	putative ribonuclease HI	0.074 (0.049, 0.099)
	chr	*bb0344*	UvrD, DNA helicase II	0.103 (0.108, 0.098)
	chr	*bb0467*	laccase domain-containing protein	0.162 (0.119, 0.205)
	lp54	*bba54*	hypothetical protein	0.243 (0.193, 0.293)
	chr	*bb0431*	CobQ/MinD nucleotide binding domain-containing protein	0.247 (0.293, 0.202)
	chr	*bb0617*	hypothetical protein	0.294 (0.295, 0.292)
	chr	*bb0267*	hypothetical protein, DUF342	0.295 (0.299, 0.292)
TBHP	chr	*bb0434*	Spo0J, Spo0J family partition protein	0.110 (0.171, 0.050)
	chr	*bb0317*	riboflavin ABC transporter permease	0.164 (0.169, 0.160)
	chr	*bb0017*	putative membrane protein, GlnB-like domain	0.231 (0.228, 0.233)
	chr	*bb0631*	hypothetical protein	0.257 (0.188, 0.326)
	chr	*bb0412*	putative membrane protein	0.291 (0.288, 0.293)
H_2_O_2_	chr	*bb0164*	putative sodium/calcium exchanger-like protein	0.069 (0.085, 0.053)
	chr	*bb0473*	putative MATE transporter	0.077 (0.056, 0.097)
	chr	*bb0202*	putative CorC-like transporter protein, CBS domain	0.094 (0.101, 0.086)
	cp26	*bbb06*	ChbB, chitobiose transporter protein	0.100 (0.108, 0.093)
	chr	*bb0669*	CheA2, chemotaxis histidine kinase	0.114 (0.033, 0.195)
	cp9	*bbc07*	hypothetical protein	0.122 (0.109, 0.134)
	lp28-2	*bbg02*	hypothetical protein	0.124 (0.127, 0.122)
	chr	*bb0803*	TruB, tRNA pseudouridine 5S synthase	0.164 (0.215, 0.113)
	lp25	*bbe29*	pseudogene	0.168 (0.146, 0.190)
	chr	*bb0025*	putative transcriptional regulator, YebC-like	0.183 (0.320, 0.046)
	lp54	*bba14*	hypothetical protein, orfD-containing	0.184 (0.169, 0.198)
	chr	*bb0243*	GlpD, glycerol-3-phosphate dehydrogenase	0.205 (0.234, 0.176)
	chr	*bb0363*	PdeA, c-di-GMP phosphodiesterase	0.224 (0.234, 0.215)
	lp36	*bbk52*	protein P23	0.235 (0.210, 0.260)
	chr	*bb0412*	putative membrane protein	0.249 (0.297, 0.201)
	chr	*bb0017*	putative membrane protein, GlnB-like domain	0.256 (0.265, 0.247)
	lp36	*bbk48*	immunogenic protein P37	0.262 (0.257, 0.267)
	chr	*bb0347*	fibronectin binding protein	0.263 (0.300, 0.226)
	chr	*bb0829*	SbcD, exonuclease	0.265 (0.260, 0.269)
	chr	*bb0556*	carbon monoxide dehydrogenase subunit-like protein	0.274 (0.278, 0.271)
	chr	*bb0638*	NhaC2, sodium/proton antiporter	0.284 (0.323, 0.245)
	chr	*bb0631*	hypothetical protein	0.301 (0.304, 0.298)

Chr, chromosome

**Fig 3:**
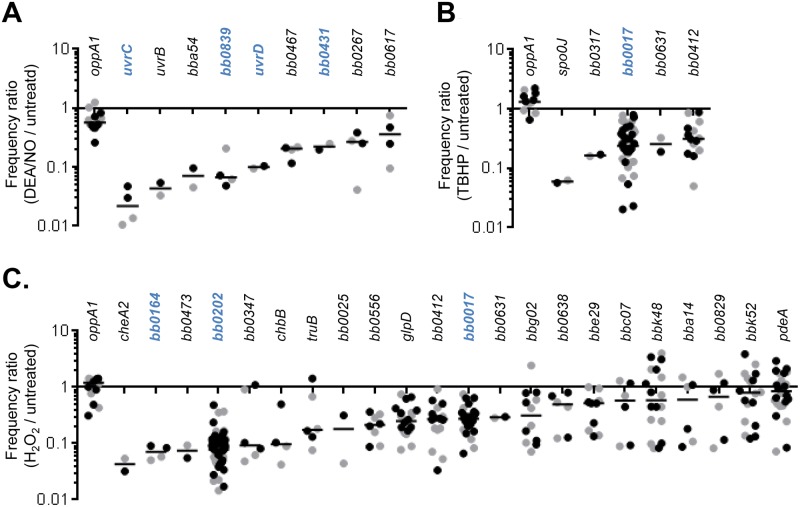
Frequency ratios of individual Tn mutants with insertions in putative ROS and RNS resistance genes. We prioritized genes for further study if they had overall frequency ratios <0.33 in both replicates after exposure to DEA/NO (A), TBHP (B), or H_2_O_2_ (C). The frequency ratios of individual Tn mutants with insertions in these genes are shown for both replicate 1 (black circles) and replicate 2 (gray circles). The median is indicated with a bar. Genes that were selected for further analysis are shown in blue.

Having selected genes of interest based on the overall frequency ratio at the gene level, we next looked at the phenotypes of the individual Tn mutants with insertions in these genes. In all cases, the frequency ratios for individual Tn mutants were similar between mutants and across replicates (Fig 3A). We decided to focus on the genes *bb0839* and *bb0431*. We selected two Tn mutants with insertions in each of these genes from the arrayed library. We also selected two Tn::*uvrC* mutants, two Tn::*uvrD* mutants, and one Tn::*oppA1* mutant for use as controls. The *oppA1* gene encodes an oligopeptide permease, and Tn mutants with insertions in *oppA1* had neutral frequency ratios (Fig 3A). All individual transposon mutants were screened by PCR at the appropriate locus, and clonal isolates were derived if a mixed population was detected. *B*. *burgdorferi* strain 5A18NP1 has a segmented genome containing 19 linear and circular plasmids, some of which are required for survival in mice and ticks [36]. We therefore determined the plasmid profiles of all the individual Tn mutants by multiplex PCR (S5 Table) [37].

The ability of the various Tn mutants to grow in semi-solid agarose overlays differed, making it difficult to assess percent survival by plating for colony forming units as we had done with the parental strain. Instead, we designed an outgrowth assay in liquid culture to assess the sensitivity of the Tn mutants to NO. A similar assay was used previously to assess the susceptibility of *B*. *burgdorferi* to UV and NO exposure [34]. Individual Tn mutants were incubated in the presence or absence of DEA/NO in parallel with the parental strain 5A18NP1. The cultures were then diluted five-fold and allowed to grow until the untreated parental strain reached late log phase (~1x10^8^ cells/ml, requiring three days). Genomic equivalents were quantified by qPCR for *recA*, and an outgrowth ratio was determined for each strain by comparing genomic equivalents in the treated and untreated samples.

**Fig 4:**
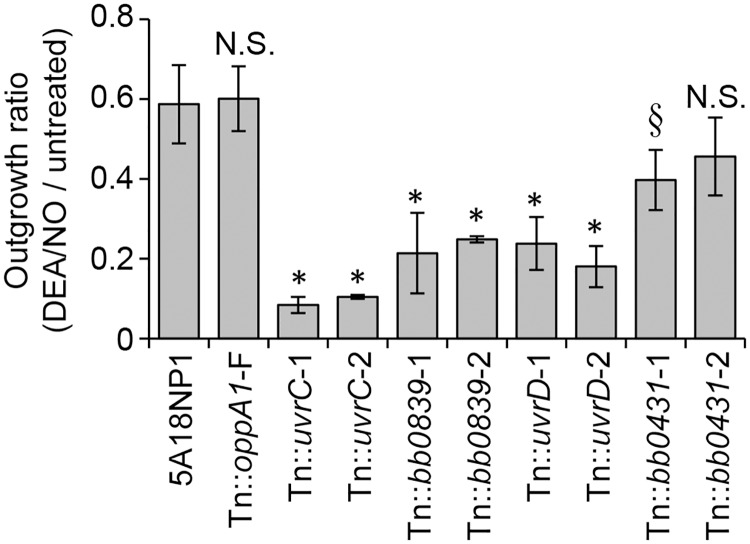
Confirmation of NO sensitivity. Individual Tn mutants were exposed to 1.25 mM DEA/NO or culture medium alone along with the parental strain 5A18NP1. Genomic equivalents were quantified following a three-day outgrowth period, and an outgrowth ratio was determined for each strain as the ratio of genomic equivalents in the treated sample compared to the untreated sample. See S5 Table for Tn clone numbers. *, P < 0.01 compared to 5A18NP1; §, P < 0.05 compared to 5A18NP1 by 1-way ANOVA followed by Dunnett’s test. N.S., not significant.

The outgrowth ratio for a Tn::*oppA1* mutant was not significantly different from the parental strain, while the Tn::*uvrC* and Tn::*uvrD* mutants displayed significantly reduced outgrowth (Fig 4). Two Tn::*bb0839* mutants also showed decreased outgrowth relative to the parental strain (Fig 4). The fact that two individual Tn mutants with insertions in the same gene both display an outgrowth defect decreases the likelihood that the phenotype is due to a second-site mutation. However, since *bb0839* is the second gene in a predicted three-gene operon, we cannot rule out potential polar effects of the transposon insertion. The downstream gene (*bb0838a*) encodes a short 182 amino acid protein annotated as a hypothetical protein, but there are no Tn mutants with insertions in this gene in the library. We also tested two Tn::*bb0431* mutants and observed a more modest phenotype compared to the Tn::*bb0839* mutants (Fig 4). Only one of the mutants reached statistical significance (Fig 4). Treatment of the Tn::*bb0839* and Tn::*bb0431* mutants with diethylamine (DEA) had no effect on bacterial growth, confirming that the sensitivity of these mutants to DEA/NO was due to the release of NO and not due to sensitivity to the DEA backbone (S2 Fig).

### Validation of putative ROS resistance genes

We next focused on genes with overall frequency ratios < 0.33 in both replicates following TBHP or H_2_O_2_ exposure (five in the case of TBHP and 22 in the case of H_2_O_2_) (Table 1). Three genes (*bb0017*, *bb0412*, and *bb0631*) had overall frequency ratios < 0.33 in both the H_2_O_2_ and TBHP screens, and all three encode predicted inner membrane proteins. The other genes with overall frequency ratios < 0.33 after TBHP treatment were *spo0J*, encoding a chromosome segregation protein, and *bb0317*, which encodes the permease subunit of a riboflavin ABC transporter previously shown to be involved in H_2_O_2_ resistance (Table 1) [23]. The H_2_O_2_ screen identified more genes than the TBHP screen, and the strongest phenotypes were for Tn mutants with insertions in genes encoding predicted transmembrane proteins or transporters: *bb0164*, *bb0473*, *bb0202*, and *chbB* (Table 1). The *bb0164* gene encodes a member of the Ca^2+^:cation antiporter (CaCA) family; *bb0473* encodes a putative multidrug and toxin compound extrusion (MATE) transporter; *bb0202* encodes a putative CorC-like Mg^2+^/Co^2+^ transporter protein; and *chbB* encodes a chitobiose transporter protein [38]. In addition to genes encoding hypothetical proteins with no known function (*bba14*, *bbc07*, *bbe29*, *bbg02*, *bbk48*, and *bbk52*), we also identified a gene encoding a putative transcriptional regulator (*bb0025*), glycerol-3-phosphate dehydrogenase (*glpD*), cyclic-di-GMP phosphodiesterase (*pdeA*), a fibronectin binding protein (*bb0347*), a histidine kinase involved in chemotaxis (*cheA2*), a carbon monoxide dehydrogenase subunit-like protein (*bb0556*), a predicted sodium/proton antiporter (*bb0638*), tRNA pseudouridine synthase (*truB*), and an exonuclease (*sbcD*) [20, 35, 39–42].

As described above, we prioritized genes with the lowest overall frequency ratios and examined the frequency ratios of individual Tn mutants with insertions in these genes (Fig 3B and 3C). In most cases, the genes identified in the TBHP and H_2_O_2_ screens were disrupted in multiple Tn mutants, although in some cases the frequency ratios of the individual Tn mutants were variable (Fig 3B and 3C). We decided to focus on *bb0017*, which was identified in both the H_2_O_2_ and TBHP screens, as well as *bb0202* and *bb0164*, which had two of the lowest overall frequency ratios in the H_2_O_2_ screen (Table 1). Two of these genes were well-represented by individual Tn mutants in the library (approximately 20 Tn::*bb0017* mutants and 40 Tn::*bb0202* mutants, Fig 3). Furthermore, the frequency ratios of the individual Tn mutants with insertions in these genes were similar to one another and similar across replicates (Fig 3B and 3C). We obtained individual Tn mutants with insertions in *bb0017*, *bb0164*, and *bb0202* from the arrayed Tn library, derived clonal stocks if necessary, and determined the plasmid profile, as described above (S5 Table).

**Fig 5:**
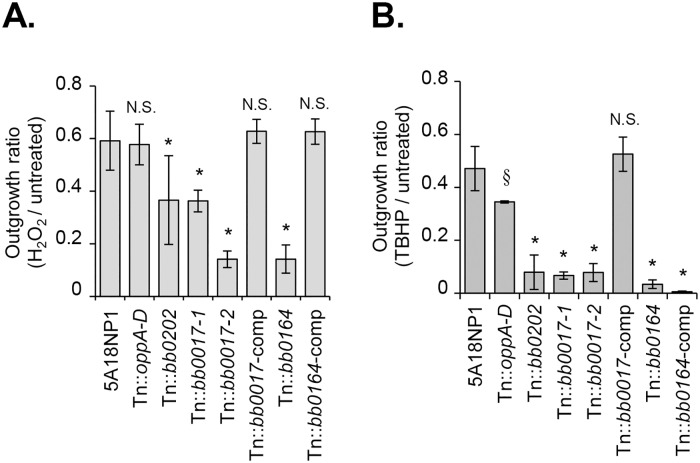
Confirmation of ROS sensitivity. Individual Tn mutants, complemented mutants, and the parental strain were exposed to H_2_O_2_, TBHP, or culture medium alone. Bacterial cell density was quantified by dark-field microscopy following a three-day outgrowth period. An outgrowth ratio was determined for each strain as the ratio of cell numbers in the treated sample compared to the untreated sample. Results for 125 μM H_2_O_2_ (A) or 7.5 mM TBHP (B) are shown. *, P < 0.01 compared to 5A18NP1; §, P < 0.05 compared to 5A18NP1 by one-way ANOVA followed by Dunnett’s test. See S5 Table for Tn clone numbers and S6 Table for a description of the complemented Tn mutants (Tn::*bb0017*-comp, DM104; Tn::*bb0164*-comp, JH511).

We then tested the sensitivity of the Tn mutants to TBHP and H_2_O_2_ using an *in vitro* outgrowth assay similar to the one described above. Tn mutants with insertions in *bb0017*, *bb0164*, and *bb0202* displayed decreased outgrowth relative to the parental strain after both TBHP and H_2_O_2_ exposure (Fig 5). The outgrowth ratio of the Tn::*oppA1* mutant, included as a negative control, was similar to the parental strain after H_2_O_2_ or TBHP exposure (Fig 5).

To verify that the disrupted genes were required for ROS sensitivity, we performed genetic complementation in an attempt to restore expression of the disrupted genes in the Tn::*bb0017*, Tn::*bb0164*, and Tn::*bb0202* mutants. The *bb0017* gene is not part of a predicted operon. Since the Tn insertion in *bb0017* is not predicted to have polar effects, complementation of *bb0017* was performed in *cis*. The Tn-disrupted locus was replaced with a full-length copy of *bb0017* as well as a streptomycin resistance marker by allelic exchange, generating strain Tn::*bb0017*-comp (S6 Table, DM104). We confirmed that *bb0017* expression was restored in the complemented strain by reverse transcriptase PCR (S3 Fig). The H_2_O_2_ and TBHP resistance of the Tn::*bb0017*-comp strain was comparable to the parental strain, confirming that the phenotype of the Tn::*bb0017* mutant was due to disruption of *bb0017* (Fig 5). *bb0164* is the second gene of a predicted operon, so complementation was performed at an unlinked site in the chromosome. A full-length copy of *bb0164* linked to its predicted native promoter was introduced at a distant chromosomal site between the genes *bb0445* and *bb0446* along with a streptomycin resistance marker, generating strain Tn::*bb0164*-comp (S6 Table, JH511) [43]. Complementation of *bb0164* restored parental levels of resistance to H_2_O_2_ but not TBHP (Fig 5). The reason for this partial complementation is unclear. The gene downstream of *bb0164* (*bb0163*) had a neutral overall frequency ratio after TBHP and H_2_O_2_ exposure, suggesting that the ROS-sensitivity of the Tn::*bb0164* mutant is not due to polar effects on the downstream gene (S1 and S2 Tables). Although the phenotype of the Tn::*bb0202* mutant was consistent with its ROS sensitivity in the Tn-seq experiments, our attempts to complement this gene have not been successful.

### BB0164 affects intracellular manganese levels in *B*. *burgdorferi*

**Fig 6:**
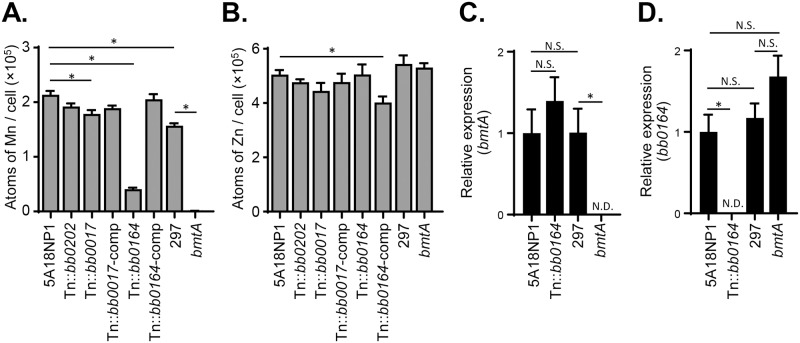
Mn levels are decreased in the Tn::*bb0164* mutant compared to the parental strain. Cellular levels of Mn (A) or Zn (B) were determined using ICP-MS in the 5A18NP1 parental strain as well as in several Tn mutants and their respective complemented strains. Mn levels were also measured in the *bmtA* mutant and its parental 297 strain [22]. All strains were grown to early stationary phase in BSK-II medium. Data represent the mean ± standard deviation of three independent experiments. *, P<0.0001 by one-way ANOVA followed by Tukey’s test. Quantitative reverse transcriptase PCR (RT-qPCR) was used to measure expression levels of *bmtA* (C) and *bb0164* (D) in the 5A18NP1, Tn::*bb0164*, 297, and *bmtA* strains. *, P<0.001 by one-way ANOVA followed by Tukey’s test. N.S., not significant. N.D., not detected. See S6 Table for a description of the complemented Tn mutants (Tn::*bb0017*-comp, DM104; Tn::*bb0164*-comp, JH511).

Given that *bb0017*, *bb0164*, and *bb0202* all encode predicted membrane proteins and that *bb0164* and *bb0202* are specifically predicted to encode transporters of divalent cations, we used inductively coupled plasma mass spectrometry (ICP-MS) to investigate intracellular levels of transition metals in these mutants. We hypothesized that perturbations in cellular levels of manganese (Mn), zinc (Zn), iron (Fe), or copper (Cu) in the Tn::*bb0017*, Tn::*bb0164*, or Tn::*bb0202* mutants could be responsible for their altered ROS sensitivity, as is the case for the previously studied *bmtA* or *bicA* mutants [19, 22]. Strikingly, the Tn::*bb0164* mutant exhibited an approximately 80% reduction in Mn levels compared to the parental 5A18NP1 (Fig 6A). Mn levels were restored to parental levels in the complemented strain (Tn::*bb0164*-comp, Fig 6A). As a control, we measured Mn levels in the *bmtA* mutant along with the 297 parental strain and, in agreement with a previous study [22], saw an almost complete reduction in Mn levels in the *bmtA* mutant (Fig 6A). Mn levels in the 297 background were slightly but significantly reduced relative to the 5A18NP1 background (Fig 6A). Zn levels were generally similar between the strains, with only the Tn::*bb0164*-comp strain exhibiting a slight but significant reduction in Zn (Fig 6B). There were no significant differences in iron or copper levels amongst the different strains (*P*>0.13 by two-way ANOVA test followed by Dunnett’s multiple comparison test).

Given the fact that *bb0164* and *bmtA* both appear to affect Mn levels, it was striking that mutation of either gene individually would lead to a reduction in intracellular Mn. To test whether the reduced levels of Mn in the Tn::*bb0164* mutant were due to an indirect effect on *bmtA* expression or vice versa, we used quantitative reverse transcriptase PCR to measure *bb0164* and *bmtA* expression in the different mutant and parental strains. Expression of *bmtA* was not significantly different in the Tn::*bb0164* mutant compared to the two parental strains, and the same was true of *bb0164* expression in the *bmtA* mutant (Fig 6C and 6D).

### Many putative ROS and RNS resistance genes are important during murine infection

Having identified a number of genes involved in RNS and ROS resistance *in vitro*, we asked whether any of these genes are required during the murine stage of the *B*. *burgdorferi* lifecycle. We generated a mini-library of 39 Tn mutants, broadening our criteria to include Tn mutants that changed more than two-fold in frequency in any of our screens. Many of these genes have not been previously studied in the context of murine pathogenesis. All of the Tn mutants were obtained from the arrayed library, clonal populations were derived if necessary, and the plasmid profiles were determined (S5 Table). The Tn mutants included in the library contained all plasmids necessary for murine infection (lp25, lp28-1, and lp36), with two exceptions (T09TC420, one of two Tn::*bb0017* mutants, and T07TC421, one of two Tn::*uvrD* mutants) [36, 44]. The Tn mutants were pooled such that each mutant was present in approximately equal numbers. In addition to 36 Tn mutants with insertions in genes of interest, the mini-library also included two Tn::*oppA1* mutants and one Tn::*pncA* mutant as positive and negative controls, respectively. The *oppA1* gene is not needed for murine infection [45], while *pncA* is required [46]. The two Tn::*oppA1* mutants were added to the library at twice the concentration of the other mutants to more closely mimic the conditions of the full library screen where a large proportion of mutants displayed neutral phenotypes.

**Fig 7:**
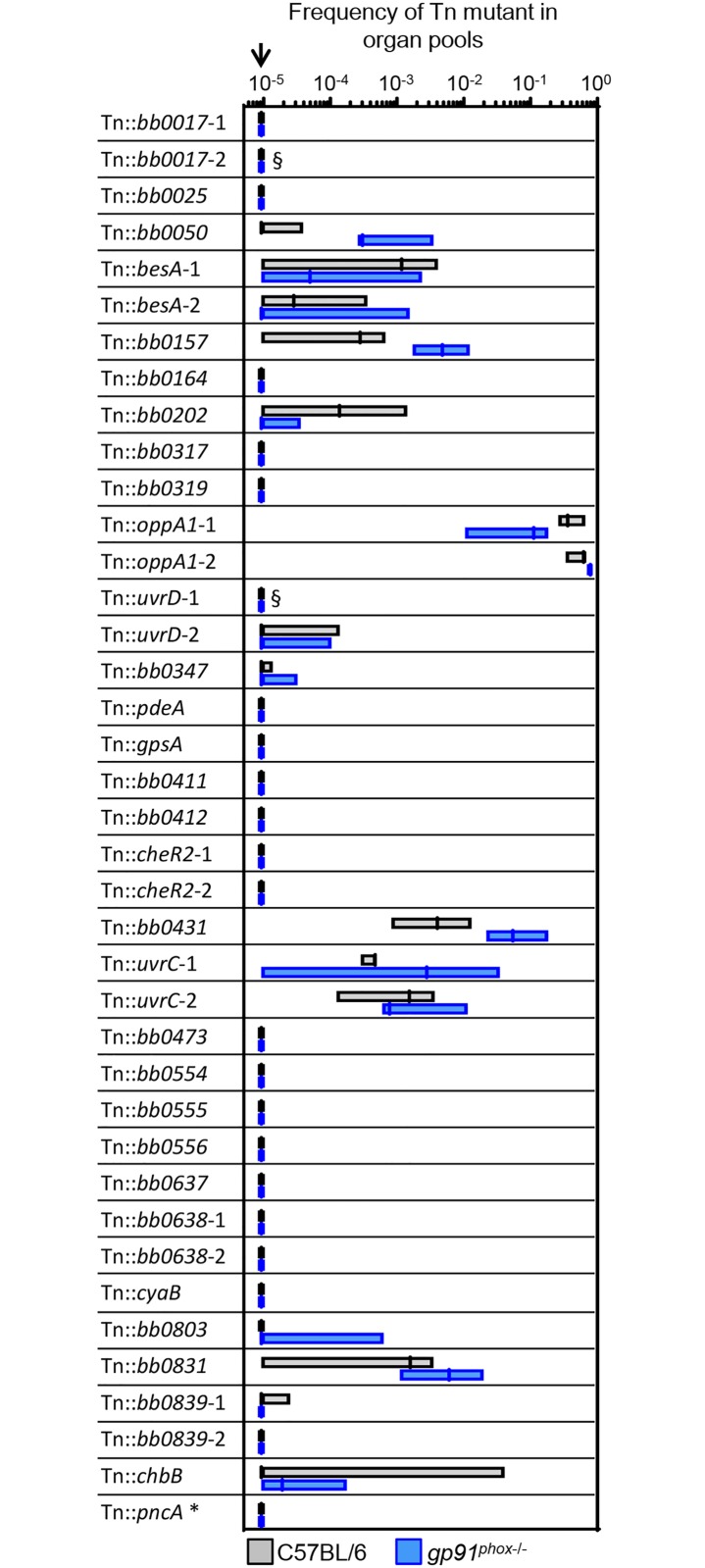
Infectivity of 39 Tn mutants in C57BL/6 and *gp91*^phox-/-^ mice. Three groups of six C57BL/6 and three groups of five or six *gp91*^*phox*-/-^ mice were infected with a mini Tn library containing 39 Tn mutants at a dose of 1×10^5^ bacteria. Pools of organs were collected from each group of mice and cultured in BSK-II medium to expand the *B*. *burgdorferi* population. The frequency of each Tn mutant in the three C57BL/6 organ pools and the three *gp91*^*phox*-/-^ organ pools was determined using Tn-seq. The boundaries of each box indicate the minimum and maximum values, with the median indicated by a bar. §, mutants are missing plasmids important for murine infection. T09TC420 (Tn::*bb0017*-2) is missing lp28-1. T07TC421 (Tn::*uvrD*-1) is missing lp36. Thus, the effects of the Tn insertion in these two mutants cannot be discerned. The arrow indicates the limit of detection of the assay. *, the Tn::*pncA* mutant included in the mini-library was subsequently found to contain a second Tn insertion in the *hk1* gene. Hk1 is not required for murine infection [47].

We used the mini-library to infect three groups of six wild-type C57BL/6 mice as well as three groups of five or six *gp91*^*phox*-/-^ mice, which lack phagocyte superoxide production. We hypothesized that some of the Tn mutants in the library would be attenuated in C57BL/6 mice and that, if the disrupted gene was involved in ROS resistance *in vivo*, some of these mutants might be rescued for infectivity in the *gp91*^*phox*-/-^ mice. After two weeks, the mice were sacrificed, and the bladder, ankles, and ears were collected under aseptic conditions. Pools of organ types were cultured in BSK-II medium to expand the *B*. *burgdorferi* population. The initial inoculum was also cultured for a comparable period of time in BSK-II to control for any changes in Tn mutant frequency due to *in vitro* growth. Genomic DNA was isolated and pooled to generate samples representing all organs collected from the six mice in each group. By pooling DNA from multiple organ sites and mice, we aimed to minimize the effects of the significant bottleneck faced by *B*. *burgdorferi* at the inoculation site [45]. We then determined the frequency of each Tn mutant in the inocula as well as in the C57BL/6 and *gp91*^*phox*-/-^ organ pools. The frequencies of the Tn mutants were largely consistent between the different inocula (S1 Fig). The frequency of the Tn mutants in the C57BL/6 and *gp91*^*phox*-/-^ organ pools is shown in Fig 7.

Reproducibility was good between the organ groups (S1 Fig, Spearman correlation coefficients R > 0.65). Twenty-three of the 39 Tn mutants in the mini-library were present at frequencies below the limit of detection in the C57BL/6 organ pools, including the Tn::*pncA* mutant and both of the Tn mutants known to be lacking plasmids required for infectivity (Fig 7). The two Tn::*oppA1* mutants represented the overwhelming majority of the bacteria isolated from the organ pools (Fig 7, over 97%) and were the only Tn mutants in the population that increased in frequency in the organ pools relative to the inocula. On average, the two Tn::*oppA1* mutants together constituted 17 ± 0.5% of the inoculum, while the other Tn mutants each constituted <13% (median prevalence of 1.5%). Tn mutants with insertions in the NO-resistance genes *uvrD*, *uvrC*, and *bb0839* were all attenuated to some degree in the C57BL/6 mice. The Tn::*uvrD* and Tn::*bb0839* mutants were the most attenuated, present at frequencies only barely above the limit of detection, while the Tn::*uvrC* and Tn::*bb0431* mutants were present at slightly higher frequencies (representing an average of 0.2% and 0.6% of the population, respectively, Fig 7). Tn mutants with insertions in the ROS resistance genes *bb0017*, *bb0164*, and *bb0202* were also attenuated *in vivo*, with the Tn::*bb0017* and Tn::*bb0164* mutants present at frequencies below the limit of detection (Fig 7).

Interestingly, the twenty-three mutants that were completely non-infectious in the C57BL/6 organ pools remained below the limit of detection in the *gp91*^*phox*-/-^ organ pools (Fig 7). As was the case with the C57BL/6 organ pools, the two Tn::*oppA1* mutants represented the majority of the population isolated from the *gp91*^*phox*-/-^ organ pools (over 87%, Fig 7). There were three Tn mutants that showed a slight increase in frequency in the *gp91*^*phox*-/-^ organ pools relative to the C57BL/6 mice (Tn::*bb0050*, Tn::*bb0157*, and Tn::*bb0431*), but the differences were not statistically significant (Fig 7).

## Discussion

The *B*. *burgdorferi* Tn library represents a powerful tool for conducting global unbiased studies in *B*. *burgdorferi*, an organism for which genetic tools have been limited [30–32, 45, 48]. In this study, we used Tn-seq to identify genes involved in RNS and ROS resistance *in vitro*. The genes identified in the screens include both known ROS and RNS resistance factors as well as novel putative resistance factors (Table 1). Using individual Tn mutants from the arrayed library, we showed that mutation of *bb0839* resulted in increased NO sensitivity, while mutation of *bb0017*, *bb0164*, or *bb0202* resulted in increased ROS sensitivity (Figs 4 and 5). We also showed that mutation of *bb0164* affects intracellular Mn levels in *B*. *burgdorferi* (Fig 6). *In vivo* Tn-seq screens showed that many of the genes identified in the *in vitro* screens are important *in vivo*, although none of the mutants included in the mini-library were rescued for infectivity in mice lacking phagocyte superoxide. Since many of the genes disrupted in the mini-library have not been previously studied in the context of murine infectivity (approximately 40%), this study represents a significant contribution to our understanding of borrelial pathogenesis.

The four genes that we identify as being involved in RNS or ROS resistance (*bb0839*, *bb0017*, *bb0164*, and *bb0202*) have not been functionally characterized in *B*. *burgdorferi*. Interestingly, we found that loss of BB0164 results in decreased intracellular Mn levels. BB0164 is annotated as a potassium-dependent sodium/calcium exchanger and is part of the larger CaCA transporter superfamily. CaCA family transporters are found in both eukaryotes and prokaryotes and have been shown to transport a variety of ions in addition to Ca^2+^ [49]. Secondary structure predictions and sequence alignment with other CaCA family members suggest that BB0164 maintains the 10-transmembrane domain topology typical of transporters in this family as well as many of the conserved residues involved in ion coordination (S4 Fig) [50–53]. To date, BB0164 is the second protein that has been shown to affect Mn levels in *B*. *burgdorferi*. Deletion of *bmtA*, a member of the ZIP (zinc- and iron-regulated metal protein) transporter family, also results in reduced Mn levels and increased sensitivity to ROS [22]. Interestingly, neither BB0164 nor BmtA belong to one of the three major classes of Mn transporters that have been previously described in prokaryotes, which include Nramp (natural resistance associated macrophage protein-like) family transporters, ATP-binding cassette Mn permeases, and P-type ATPase Mn transporters [22, 54], suggesting that *B*. *burgdorferi* has evolved unique strategies for Mn acquisition.

Mn has been shown to protect against oxidative stress in a wide range of organisms besides *B*. *burgdorferi* and to do so via both protein-dependent and protein-independent mechanisms [55]. In terms of protein-dependent mechanisms, Mn can serve as a co-factor for ROS detoxification enzymes such as catalases and superoxide dismutases [55]. The sole superoxide dismutase (SodA) in *B*. *burgdorferi* contains a Mn co-factor, and the reduced Mn levels of the *bmtA* mutant result in decreased SodA activity [56, 57]. The ROS sensitivity of the Tn::*bb0164* mutant in our assays is likely not due to decreased SodA activity, however, since Tn::*sodA* mutants present in the Tn library were not more sensitive to H_2_O_2_ in our initial Tn-seq screens (S1 and S2 Tables). This is perhaps not surprising, since SodA catalyzes the dismutation of superoxide into molecular oxygen and H_2_O_2_, and a *sodA* mutant would not be predicted to display increased sensitivity to the product of its reaction. In addition to protein-dependent mechanisms of protection, Mn can also directly scavenge ROS and can form non-proteinaceous complexes with small metabolites in the cell [55]. These so-called Mn-antioxidants are important for ROS resistance in organisms such as *Lactobacillus plantarum* [58]. Like *L*. *plantarum*, *B*. *burgdorferi* accumulates high levels of Mn [19, 57], and it is possible that Mn-antioxidants also contribute to ROS resistance in *B*. *burgdorferi*.

Loss of either *bb0164* or *bmtA* expression results in decreased Mn levels (Fig 6A). These data suggest that BB0164 and BmtA are not redundant with one another and that the presence of both proteins is required for maintaining Mn homeostasis (Fig 6A) [22]. The mechanism by which BB0164 and BmtA together affect intracellular Mn levels remains unclear. The two proteins could form a complex necessary for Mn transport or mediate sequential steps in the same pathway. Although we showed that *bb0164* expression was not significantly different in the *bmtA* mutant and vice versa (Fig 6C & 6D), it remains possible that loss of BB0164 or BmtA reduces the stability or steady state levels of the remaining protein. Finally, although inactivation of either BB0164 or BmtA decreases cellular Mn levels and both proteins contain sequence and structure motifs consistent with a role in cation transport (S4 Fig) [22], there is no direct biochemical evidence that either protein is a *bona fide* Mn transporter. Control of metal homeostasis in the cell is a complex process, and it remains possible that only one of these proteins is directly responsible for the transport of Mn, while the other has an indirect effect on Mn levels via transport of an alternative substrate.

The mechanisms by which *bb0839*, *bb0017*, and *bb0202* provide resistance against ROS or RNS require further investigation. BB0839 is annotated as a hypothetical protein, but protein structure predictions suggest that it is similar to RNase HI, a nonspecific RNA endonuclease that seems to be unrelated to RNS protective activities [35]. Therefore additional verification (e.g. isolation of additional mutants and/or complementation) of its potential role in protection against RNS is needed. The two remaining genes encode predicted transmembrane proteins. BB0202 contains an N-terminal predicted transmembrane region and a C-terminal domain with tandem repeats of a cystathionine beta-synthase (CBS) domain. Other proteins with similar domains are involved in ion transport. A structure-based analysis of BB0202 using Phyre^2^ suggested that it is similar to CorC, a protein involved in magnesium and cobalt efflux in *Salmonella typhimurium* [35]. BB0017 is annotated as an integral membrane protein [20]. It contains a predicted N-terminal transmembrane domain as well as a C-terminal domain containing two conserved domains of unknown function (DUF161 and DUF2179). While BB0017 and BB0202 are apparently not involved in the transport of Mn or Zn (Fig 6A and 6B), these proteins could be involved in transport of other small molecules or ions. Given that *B*. *burgdorferi* encodes relatively few canonical oxidative stress response proteins and a significant portion of the genes identified in the ROS Tn-seq screens encode predicted membrane proteins, we hypothesize that transport serves as an alternative detoxification strategy in *B*. *burgdorferi*. Transported substrates could provide protection from oxidative stress by directly scavenging ROS, by providing co-factors for detoxification enzymes, or by promoting osmoregulation in the face of membrane damage. The transport of small molecules such as polyamines, cystine, and glutathione affects sensitivity to oxidative stress in a variety of organisms [59–62]. With the exception of several studies regarding the transport of Mn and riboflavin [22, 63], very little is known about the transport of small molecules and ions in *B*. *burgdorferi*.

While *bb0839*, *bb0017*, *bb0164*, and *bb0202* are the only genes that we validated as protective against ROS and RNS, the Tn-seq screens identified 66 genes that had an overall frequency ratio below our neutral fitness cut-off value of 0.5 (S3 Table). Almost half of these genes encode proteins of unknown function (Fig 2), highlighting the usefulness of unbiased high-throughput genetic screens like Tn-seq for imputing functions to uncharacterized proteins. The genes identified in the DEA/NO screen were largely different from those identified in the H_2_O_2_ and TBHP screens, suggesting that the cellular responses to RNS and ROS are different (Fig 2A). In contrast, there was significant overlap between the genes identified in the TBHP and H_2_O_2_ screens (Fig 2A). Since TBHP induces lipid peroxidation and membrane damage is one effect of ROS toxicity in *B*. *burgdorferi* [13], we hypothesize that the genes identified in both screens may encode proteins involved in a membrane damage response. In addition to *bb0017* and *bb0412*, these genes include *bb0050* and *bb0051* (encoding putative energy coupling factor transporters), *hk1* (encoding histidine kinase 1), *bb0554*, *bb0555*, and *bb0556* (encoding proteins with limited homology to the subunits of a carbon monoxide dehydrogenase), *bb0631* (encoding a putative lipoprotein), and *bbe29* (a pseudogene). Of this list, Hk1 is particularly intriguing since it is a histidine kinase which, along with the response regulator Rrp1, constitutes one of the two-component systems in *B*. *burgdorferi* [47, 64]. The Hk1/Rrp1 two-component system regulates synthesis of cyclic di-GMP, controlling a variety of cellular processes that are required for bacterial survival of the larval and nymphal blood meals [64]. The identification of *hk1* in this screen suggests that one facet of the Hk1/Rrp1 response may contribute to ROS resistance. We compared our list of 66 genes with those from previous studies on gene expression and regulator binding sites in *B*. *burgdorferi* and generally saw little or no overlap. Strikingly, however, 23 of the 66 genes are located downstream of one of the 156 putative BosR binding sites identified in a recent study [29], suggesting that the expression of these genes could be coordinately regulated.

Although we focused on Tn mutants that decreased in frequency after stress exposure, there were a few cases where Tn mutants significantly increased in frequency, suggesting that disruption of these genes makes *B*. *burgdorferi* more resistant to ROS or RNS (S4 Table). The phenotype was particularly striking for Tn mutants with insertions in *bb0637* and *bb0638*, which had overall frequency ratios of 7.3 and 10.2 in the TBHP screen (S4 Table). BB0637 and BB0638 are annotated as putative Na^+^/H^+^ antiporters of the ArsB/NhaD superfamily but have not been functionally characterized in *B*. *burgdorferi*. The phenotype we observe for the Tn::*bb0637* and Tn::*bb0638* mutants is similar to that reported for the *bicA* mutant, which is more resistant to peroxide stress compared to the parental strain when grown in iron- or copper-replete media [19]. It was hypothesized that although BicA-mediated metal sequestration prevents the metal from binding to and damaging other proteins, BicA also effectively increases the cellular metal concentration and does not prevent reactivity with hydrogen peroxide to form hydroxyl radical [19]. Based on the results of our *in vivo* Tn-seq screen, *bb0637* and *bb0638* appear to play essential roles *in vivo* (Fig 7), and we hypothesize that ROS sensitivity may be an unavoidable consequence of this essential BB0637 and BB0637 function.

A potential caveat to Tn library-based screens in *Borrelia* is the segmented nature of the genome. Strain B31 5A18NP1 contains 19 of 21 borrelial linear and circular plasmids in addition to the linear chromosome [65]. Although several of the plasmids are required for infectivity in mice and ticks, the lack of a selective pressure during growth *in vitro* can result in plasmid loss. The plasmid profile has been determined for 4,464 Tn mutants in the arrayed library [30]. Although we minimized *in vitro* passage of the library, it is possible that plasmid loss could have occurred after the arrayed mutants were pooled. As a result, phenotypes detected in the Tn-seq screens could be due to plasmid loss instead of the Tn insertion. However, it is unlikely that plasmid loss significantly affects our overall conclusions. The majority of the genes identified in our *in vitro* screens are chromosomally encoded, suggesting that plasmid-encoded genes have a limited contribution ROS and RNS resistance. Additionally, many of the genes with the lowest overall frequency ratios in our screens were disrupted in multiple Tn mutants, making it unlikely that plasmid loss accounts for their sensitivity to ROS or RNS. We also independently confirmed the plasmid profile for each individual Tn mutant we obtained from the arrayed library (S5 Table), confirmed the phenotypes for four of these Tn mutants independently *in vitro*, and were able to complement the phenotype in two of the Tn mutants (Fig 5).

All 36 Tn mutants selected based on the results of our *in vitro* Tn-seq screens exhibited a competitive defect relative to the neutral Tn::*oppA1* mutants *in vivo*, and more than half of the mutants in the library were undetectable in our assay following infection (Fig 7). Many of these gene products have not been functionally characterized and are thus interesting candidates for future study. In cases where genes disrupted in our mini-library have been tested previously for their roles *in vivo*, our results generally agree with those from previous studies, further validating the use of Tn-seq for *in vivo* studies of *B*. *burgdorferi* pathogenesis [30, 32]. For example, earlier signature tagged mutagenesis or Tn-seq screens included Tn mutants with insertions in 19 of the same genes as the Tn mutants included in our mini-library; 17 of these 19 mutants had at least partial infectivity defects in both studies [30, 32]. Several of the mutants included in our mini-library have also been tested in single strain infections (for example, *uvrC*, *uvrD*, *pdeA*, and *cyaB*) [34, 41, 66]. Again, our results are largely consistent with the results of these previous studies, and differences in the nature of single-strain versus competition experiments could explain some minor discrepancies [66].

The results of the *in vivo* Tn-seq screen in C57BL/6 mice suggest that the ability to detoxify ROS or RNS is important for *B*. *burgdorferi* pathogenicity. Interestingly, however, none of the Tn mutants were rescued for infectivity in the *gp91*^*phox-/-*^ mice as we had hypothesized (Fig 7). Our findings are similar to those of a recent study showing that the infectivity of a *sodA* mutant, which lacks the sole borrelial superoxide dismutase, is not rescued in the *gp91*^*phox-/-*^ background [67]. It remains unclear exactly when and where *B*. *burgdorferi* encounters ROS in the mammalian host and whether *gp91*^*phox*^ plays an important role in controlling *B*. *burgdorferi* infection. Two previous studies using *gp91*^*phox-/-*^ mice have reported, in one instance, no difference in the severity of Lyme arthritis [68], and in the other, a slight increase in the bacterial burden at several tissue sites [67]. Although one possible interpretation of our results is that the genes we have identified are not involved in ROS detoxification *in vivo*, another possible explanation is that loss of these ROS detoxification genes has secondary effects on the cell that lead to attenuation independent of ROS sensitivity. For example, reduced Mn levels in the *bmtA* mutant lead to decreased SodA activity and increased ROS sensitivity, but also result in the dysregulation of lipoproteins required for virulence [8, 22, 56, 57]. The reduction of Mn in the Tn::*bb0164* mutant presumably has similar consequences for lipoprotein expression. While the absence of superoxide in the *gp91*^*phox-/-*^ background might be predicted to compensate for the ROS sensitivity of the *bmtA* and Tn::*bb0164* mutants, dysregulated lipoprotein expression might still result in the attenuation of these strains.

Given the fact that *B*. *burgdorferi* must constantly evade the immune systems of its vertebrate and invertebrate hosts, it seems certain that the bacteria come into contact with RNS and ROS at some point during their lifecycle. In particular, it seems likely that the tick-vertebrate interface represents a site where spirochetes face ROS or RNS. Future studies should characterize the magnitude of oxidative stress at this early time-point as well as additional time points during colonization of mice and ticks. Overall, this study provides further support for Tn-seq as a powerful tool for both *in vitro* and *in vivo* studies in *B*. *burgdorferi* and identifies new gene products involved in both the defense against ROS and RNS and murine pathogenesis. The unusual biochemistry, genetics, and lifestyle of *B*. *burgdorferi* make it an excellent model for identifying novel targets that add to the bacterial arsenal against ROS- and RNS-mediated damage.

## Materials and methods

### Bacterial strains and growth conditions

*Escherichia coli* strains were grown on Luria-Bertani (LB) agar plates or in LB broth at 37°C. *B*. *burgdorferi* strains were grown in Barbour-Stoenner-Kelley II (BSK-II) medium in sealed tubes at 32°C with 1% CO_2_ in static cultures unless noted otherwise. Bacteria were exposed to ROS using the Modified BSK-II medium which does not contain pyruvate, bovine serum albumin, or rabbit serum [14]. The parental strain of the transposon library, the infectious *B*. *burgdorferi* strain 5A18NP1 [65], was used as the wild-type strain in all studies. The following antibiotics were used for *B*. *burgdorferi* when appropriate: kanamycin at 200 μg/ml, gentamicin at 40 μg/ml, and streptomycin at 50 μg/ml.

Transposon mutants were obtained from the arrayed *B*. *burgdorferi* library [30]. It is important to note that there is not perfect concordance between the Tn mutants represented in our Tn-seq dataset and the Tn mutants available in the arrayed library. This is primarily a result of four factors: (1) the exclusion of Tn mutants from the Tn-seq data analysis due to low representation in the library; (2) the loss of Tn mutants from the population when the library was originally pooled; (3) the presence of co-isolates in some of the previously described Tn mutants [32, 66]; and (4) the inclusion of Tn mutants in the pooled library for which the insertion site had not been mapped. All individual Tn mutants were screened by polymerase chain reaction (PCR) at the locus of interest to confirm pure populations. In cases where mixed populations were identified (i.e. two PCR products indicating the presence of both a WT and Tn-disrupted locus), the strain was plated for single colonies in semi-solid agarose overlays [69]. Individual colonies were then selected and re-screened to confirm pure populations. All transposon mutants were subsequently plasmid-typed to identify the loss of any plasmids required for murine or tick infection [37]. See S5 Table for a description of all individual Tn mutants used in this study. Other *B*. *burgdorferi* strains as well as plasmids used in this study are described in S6 Table. Primer sequences are listed in S7 Table.

### Complementation of Tn mutants

Plasmids for complementation of *bb0017* and *bb0164* were generated by overlap PCR as described below for each gene (S6 Table). Primers were designed with approximately 30 bp on the 5’ end for overlap, denoted in the underlined sequence of S7 Table, with the numerically assigned PCR product assembled in order into the final construct. Individual PCR fragments were amplified with PrimeSTAR GXL DNA Polymerase (Clonetech, Mountain View, CA) per the manufacturer’s instructions. An overlap PCR reaction was performed with equal volumes of the appropriate number of PCR fragments with PrimeStar GXL reagents per the manufacturer’s recommendations. The amplification program was modified as follows: (1) 94°C for 4 min; (2) 94°C for 30 sec; (3) 45°C for 30 sec; (4) 68°C for 1 min/kb of largest individual fragment; (5) repeat steps 2 through 4 ten times; (6) 94°C for 30 sec; (7) 55°C for 30 sec; (8) 68°C for 1 min/kb of overlap PCR product; repeat steps 6 through 8 30 times. Following PCR, the products were resolved by agarose gel electrophoresis and gel-purified (Zymoclean Gel DNA Recovery Kit, Zymo Research, Irvine, CA) for cloning into pCR-Blunt (ThermoFisher Scientific, Grand Island, NY) following manufacturer’s protocol. Completed complementation vectors were verified by restriction digest and dideoxy sequencing.

The *cis* complementation of *bb0017* required three PCR fragments consisting of intact *bb0017* with 746 bp upstream sequence (F1), P_*flgB*_*-aadA* for antibiotic selection (F2), and 1557 bp downstream of *bb0017* (F3) for allelic exchange into the borrelial chromosome. The *bb0017* complementation vector was designated pJH508 (S6 Table). *bb0164* is part of a predicted operon, and *cis* complementation could have a polar impact on downstream genes. Thus a *trans* complementation vector for *bb0164* was designed for allelic exchange in the borrelial chromosome between *bb0445* and *bb0446*, a site previously used for the complementation of *dps/napA/bicA* [43]. The *bb0164* complementation vector, designated pJH511, consists of five fragments: 1458 bp of the *bb0445* region (F1), 490 bp upstream of *bb0165* start codon (F2), *bb0164* from the start codon to the stop codon (F3), P_*flgB*_*-aadA* for antibiotic selection (F4), and 1449 bp of the *bb0446* region for allelic exchange (F5).

*Cis* complementation vector pJH508 was transformed into *B*. *burgdorferi* Tn::*bb0017* as previously described [69, 70], and transformants were designated DM104 (Tn::*bb0017*-comp). *Trans* complementation of Tn::*bb0164* with pJH511 resulted in *B*. *burgdorferi* strain JH511 (Tn::*bb0164*-comp). The complemented strains were screened by PCR for allelic exchange using the forward primer of fragment 1 and the reverse primer for the P_*flgB*_*-aadA* cassette for each construct (S7 Table). The borrelial plasmid composition was confirmed by PCR [71].

### Determining *B*. *burgdorferi* sensitivity to ROS and RNS

To determine the sensitivity of *B*. *burgdorferi* strain 5A18NP1 to H_2_O_2_, TBHP, and DEA/NO, the strain was grown to mid-log phase in BSK-II, and cell density was determined by dark-field microscopy. A total of 5×10^7^ bacteria were exposed to culture medium alone or to increasing concentrations of each ROS or RNS reagent in a 1-ml volume. In the case of H_2_O_2_ and TBHP, bacteria were harvested by centrifugation at 14,000 × *g* for 5 min, washed in PBS, and resuspended in Modified BSK-II prior to ROS exposure [14]. After a two-hour (for DEA/NO) or a four-hour (for H_2_O_2_ and TBHP) stress exposure, bacteria were serially diluted in fresh BSK-II, and dilutions were plated in semi-solid agarose overlays [69]. The four-hour ROS exposure was selected to conform with exposure lengths in other published studies. A shorter incubation time was chosen for the DEA/NO exposure because of the short half-life of the molecule (2 min at 37°C). Plates were incubated for 10–12 days at 32°C with 1% CO_2_ before enumerating colony forming units (CFUs). Percent survival was determined by dividing the number of CFUs on the treated plates by the number of CFUs on the media-alone control.

The sensitivity of individual Tn mutants to DEA/NO, TBHP, and H_2_O_2_ was determined using an outgrowth assay similar to one previously described [34]. Tn mutants were grown for three days in BSK-II. The cell density of the cultures was determined using dark-field microscopy, and the cultures were diluted to a concentration of 2×10^7^/ml and grown overnight. This ensured that the Tn mutants were at a similar point of their growth curve on the day of the experiment despite any differences in growth rate. Tn mutants were exposed to culture medium, 1.25 mM DEA/NO, 0.125 mM H_2_O_2_, or 7.5 mM TBHP using the conditions described above. These concentrations were empirically selected to result in approximately 60% survival of the parent strain. At the end of the stress exposure, the cells were harvested by centrifugation for 5 min at 14,000 × *g*, resuspended in 1 ml BSK-II, and diluted 5-fold in BSK-II. Cell density was quantified after three days, when the cell density of the untreated parental culture reached ~1×10^8^/ml. For the DEA/NO experiments, a 0.4-ml volume of each culture was harvested by centrifugation, and cell equivalents were quantified by qPCR for the *recA* gene. An outgrowth ratio was determined for each strain by dividing the cell equivalents in the presence of DEA/NO by the cell equivalents in the untreated culture. For the TBHP and H_2_O_2_ experiments, cell numbers were quantified using dark-field microscopy.

### *In vitro* Tn-seq

The *B*. *burgdorferi* signature tagged transposon mutagenesis library is maintained as an arrayed library [30]. In order to conduct Tn-seq experiments, a single pool containing all of the Tn mutants was generated by combining sub-pools containing 70–80 individual Tn mutants each [32]. For the current study, the Tn library was re-pooled to eliminate an 80-mutant sub-pool containing one Tn mutant (with an insertion in *bbj05*) that was often over-represented in the library. The re-pooled Tn library was grown for three days in the presence of kanamycin and gentamicin. Cell density was determined by dark-field microscopy, and 5×10^7^ cells were resuspended in 1 ml of BSK-II medium for exposure to DEA/NO. In the case of TBHP or H_2_O_2_ exposure, bacteria were harvested by centrifugation at 14,000 × *g* for 5 min, washed once in PBS, and resuspended in 1 ml Modified BSK-II. ROS and RNS reagents were added if appropriate, and the tubes were incubated for two hours (in the case of DEA/NO exposure) or four hours (in the case of ROS exposure). Bacteria were harvested by centrifugation at 6600 × *g* for 10 min. The supernatant was removed, and the bacterial pellet was resuspended in 5 ml fresh BSK-II medium containing kanamycin and gentamicin. Cultures were allowed to grow until they reached a density of approximately 1×10^8^ cells/ml, representing one log of outgrowth. Bacteria were harvested by centrifugation, and pellets were stored at -80°C until processing. Two independent experiments were performed.

### Animal studies

C57BL/6 or *gp91*^*phox*-/-^ mice were used for the *in vivo* Tn-seq experiments. To generate the mini-library for the *in vivo* Tn-seq study, 37 *B*. *burgdorferi* mutants with Tn insertions in genes of interest (S5 Table) were grown for three days in BSK-II medium containing kanamycin and gentamicin. Cell density was determined by dark-field microscopy, and equal numbers of each mutant were pooled to a final concentration of 4×10^7^ cells/ml. Aliquots of this “mini-library” were then stored at -80°C. To prepare the inoculum for infection, the mini-library and two Tn::*oppA1* mutants were grown separately in BSK-II media containing kanamycin and gentamicin for two days. Cell density was determined for each culture by dark-field microscopy, and cultures were mixed to a final concentration of 1×10^6^ cells/ml, such that the Tn::*oppA1* mutants were present at twice the concentration of the other mutants.

Three groups of six mice C57BL/6 mice and three groups of five or six *gp91*^*phox*-/-^ mice between 8–12 weeks of age were injected subcutaneously with a dose of 1×10^5^ bacteria (approximately 5×10^3^ copies of each Tn::*oppA1* mutant and approximately 2.5×10^3^ copies of each other mutant). A 100-μl aliquot of the inoculum was used to inoculate 10 ml BSK-II containing kanamycin and gentamicin. This culture was monitored daily by dark-field microscopy, and bacteria were harvested by centrifugation at 14,000 × *g* for 5 min once the cell density reached between 5×10^7^ and 1×10^8^ cells/ml. The bacterial cell pellet was frozen at -80°C. Mice were sacrificed two weeks post-infection. The bladder, both ankles, and both ears were removed under aseptic conditions. Each organ type from the six mice in the group was pooled and cultured in 10 ml BSK-II containing kanamycin and gentamicin. The cell density of the organ cultures was monitored daily, and bacteria were harvested as described above.

### Ethics statement

Mice were bred and maintained in the Tufts University Animal Facility. All experiments were performed following the guidelines of the American Veterinary Medical Association (AVMA) as well as the Guide for the Care and Use of Laboratory Animals of the National Institutes of Health. All procedures were performed with approval of the Tufts University Institutional Animal Care and Use Committee (IACUC, Protocol# B2015-159). Euthanasia was performed in accordance with guidelines provided by the AVMA and was approved by the Tufts University IACUC.

### Preparing libraries for Illumina sequencing

Genomic libraries for sequencing were constructed as described previously [45, 72], with the following changes. Chromosomal DNA was sheared using the M220 Focused-ultrasonicator (Covaris) in microTUBEs with a target peak at 350 bp. The first round of PCR amplification was performed using a modified primer with optimized annealing to the transposon (pMargent1A, 5’-ggtaccttaggagaccgggg-3’). Libraries were multiplexed and pooled for analysis. Sequencing was performed on an Illumina HiSeq 2500 at the Tufts University Core Facility as 50-bp single-end reads, as described previously [45].

### Tn-seq data analysis

Sequenced reads were split according to the barcode sequence. Data analysis was performed using the Galaxy platform [73]. We obtained an average of 1.2×10^7^ reads per barcode. Sequences were clipped to remove the C tail, and any sequences shorter than 30 bp were discarded. Sequences were then filtered by quality, and any sequences in which 95% of the base-pairs did not have a quality cut-off value (Q) greater than 30 were discarded. After this quality control work-flow, we were left with between 4×10^6^ and 5×10^6^ reads per condition. Reads were mapped to the *B*. *burgdorferi* B31 genome using Bowtie, and a custom script was used to count the number of sequence reads corresponding to each insertion site in the genome. Sequence reads were analyzed “by-site” and “by-gene.” Only transposon mutants that were represented by at least ten sequence reads in both untreated samples were included in the “by-site” analysis. In contrast, the “by-gene” analysis included all sequence reads mapping within a particular gene. Genes represented by less than ten sequence reads in both untreated samples were excluded from the “by-gene” analysis. Tn mutants with zero reads in the treated samples were assigned a value of one. The frequency of each transposon mutant in a particular barcoded condition was determined by dividing the number of sequence reads corresponding to each Tn mutant by the total number of sequences in the barcode. A frequency ratio was then determined by dividing the frequency of a transposon mutant in the ROS/RNS-exposed sample by its frequency in the untreated control. Some of the plasmids in *B*. *burgdorferi* are highly similar in sequence to one another. As a result, genes were subsequently excluded from the analysis if any of the sequence reads that mapped within the gene could be assigned to multiple locations in the genome (gray shading in S1 and S2 Tables). For the purposes of prioritizing mutants for follow-up, frequency ratios between 0.5 and 2 were considered neutral.

### Inductively Coupled Plasmid-Sector Field Mass Spectrometry (ICP-MS)

Spirochetes were grown to early stationary phase and harvested by centrifugation (4000 × *g* for 1 h at 4°C). The cells were washed three times with sterile phosphate-buffered saline (PBS, Thermo) and once with UltraPure water (Invitrogen). During the final PBS wash, the OD_600_ of a 1:20 dilution of the sample was measured to assess bacterial cell density. The cell density measurements were used to normalize metal content to cell number in the downstream analysis. The bacterial pellet was processed for ICP-MS as described previously [19]. Briefly, pellets were digested in 100 μl of concentrated nitric acid (16 N, GFS Chemicals) at 100°C for 15 min. The volume of each sample was adjusted to 1 ml by the addition of UltraPure water, and an internal indium standard was added to a final concentration of 10 ppb. Samples were analyzed at the Trace Element Research Laboratory at The Ohio State University using a PerkinElmer Nexion 350D Inductively Coupled Plasma Sector Field Mass Spectrometer, as described previously [19].

### RNA isolation

Total RNA was isolated from bacterial cells grown to mid-logarithmic growth phase using either the 5 Prime PerfectPure RNA cultured cell kit (for reverse transcriptase PCR; 5 PRIME, Inc., Gaithersburg, MD) or TRIzol (Invitrogen) following the manufacturer’s instructions. RNA samples were treated with Ambion’s TURBO DNA-*free* kit (ThermoFisher Scientific, Waltham, MA) and recombinant RNasin Ribonuclease Inhibitor (Promega Co., Madison, WI) to eliminate contaminating DNA and inhibit RNase activity, respectively.

### Reverse Transcription (RT)-PCR

Oligonucleotide primer pairs were designed for *bb0017* and for the constitutively-expressed *flaB* gene (S7 Table). The primer pairs were tested to confirm the amplification of a single product with a known size using genomic *B*. *burgdorferi* DNA as the template. Reverse transcription reactions were performed by combining Invitrogen’s SuperScript II Reverse Transcriptase (ThermoFisher Scientific, Waltham, MA) with purified RNA from the appropriate *B*. *burgdorferi* strain according to manufacturer’s instructions. The gene-specific primers *bb0017*R or *flaB*F (S7 Table) were used to synthesize cDNA. Detection of *flaB* was done to control for the isolation of RNA. A control reaction lacking reverse transcriptase was performed in all cases to test for the presence of residual DNA. Subsequently, the cDNA from the reverse transcription reactions was used as template and subjected to PCR using Takara EmeraldAmp Max PCR Master Mix (Clontech Laboratories, Mountain View, CA). To amplify the *B*. *burgdorferi* targets, the amplification program was modified as follows: (1) 94°C for 2 min; (2) 98°C for 10 sec; (3) 45°C for 30 sec; and (4) 72°C for 2 min. Steps two through four were repeated 30 times followed by a final extension step at 72°C for 10 min. The resulting samples were then resolved by agarose gel electrophoresis.

### Quantitative PCR (qPCR) analysis

Quantification of target gene expression from cDNA or from *B*. *burgdorferi* cell lysates prepared via alkaline lysis [74] was performed using the iTaq Universal SYBR Green Supermix (BioRad). cDNA was prepared using random hexamers (Promega) and the ImProm-II Reverse Transcription System (Promega). Primers used in qPCR analysis of the *recA*, *flaB*, and *bmtA* genes from *B*. *burgdorferi* have been described previously [22, 75, 76] and were added to a final concentration of 0.4 μM each (S7 Table). Cycling parameters were 95°C for 3 min followed by 39 cycles of 95°C for 10 sec, 60°C for 30 sec. Melt curve analysis was performed by increasing the temperature from 65°C to 95°C in 0.5°C increments every 5 sec. All samples were run in triplicate. A cell lysate prepared from 5×10^7^ cells of *B*. *burgdorferi* strain 5A18NP1 was used to generate a standard curve. For qPCR analysis from cDNA, samples prepared in the absence of reverse transcriptase were included to control for the presence of residual chromosomal DNA. Analysis was performed using the CFX Connect Real-Time PCR Detection System (BioRad).

## Supporting information

S1 Fig Correlation between biological replicates of the Tn-seq experiments. The frequency of each mutant in the Tn library is shown for the two biological replicates of the untreated (A-C) and treated (D-F) samples. The nonparametric Spearman correlation coefficient R is shown in each case. Also shown are the nonparametric Spearman correlation coefficients comparing each of the 6 inocula used to infect 3 groups of C57BL/6 mice and 3 groups of *gp91*^*phox*-/-^ mice with the mini-library of 39 Tn mutants (G). The frequency of each of these 39 Tn mutants in the three C57BL/6 and *gp91*^*phox*-/-^ organ pools, along with the correlation coefficients between the samples, is also shown (H-I).
(PDF)

S2 Fig Tn::*bb0431* and Tn::*bb0839* are not more sensitive to diethylamine compared to the parental strain. The Tn::*bb0431* and Tn::*bb0839* Tn mutants were exposed to 1.25 mM DEA/NO, 1.25 mM diethylamine (DEA), or culture medium alone along with the parental strain 5A18NP1. Genomic equivalents were quantified following a three-day outgrowth period, and an outgrowth ratio was determined for each strain as the ratio of genomic equivalents in the treated sample compared to the untreated sample. *, P < 0.01 compared to 5A18NP1 by 2-way ANOVA followed by Dunnett’s test. N.S., not significant.
(PDF)

S3 Fig RT-PCR of the Tn::*bb0017* strain shows the loss of the *bb0017* transcript. Total RNA was prepared from the parent 5A18NP1 (P), the Tn::*bb0017* mutant (M), and the complemented strain DM104 (C), then subjected to RT-PCR using oligonucleotide primers specific for *bb0017* (left half) and *flaB* (right half) as described in S7 Table. The absence or presence of reverse transcriptase (RT) in the samples is indicated by a minus or plus symbol, respectively. The leftmost lane contains a 100 base pair ladder with the 0.5 kb and 1 kb fragments indicated.
(PDF)

S4 Fig Alignment of BB0164 with other CaCA transporter family members. Clustal Omega was used to align the amino acid sequence of BB1064 from *B*. *burgdorferi* (Bb_BB0164) with the amino acid sequences of homologs in *Bacillus subtilis* (Bs_YfkE), *Methanococcus jannaschii* (Mj_NCX), *Saccharomyces cerevisiae* (Sc_VCX1), and *Arabidopsis thaliana* (At_CAX1). Conserved amino acids are indicated by red shading or blue borders. Numbering corresponds to the BB0164 sequence. Green rectangles indicate the 11 transmembrane domains (TM0 –TM10) identified in the crystal structure of *B*. *subtilis* YfkE (Protein Data Bank ID code 4KJS). Orange rectangles indicate predicted alpha helices in BB0164 (Phyre^2^) [35]. Amino acids that are involved in Ca^2+^ transport in *B*. *subtilis* YfkE are indicated by asterisks [50]. This figure was prepared using ESPript [77].
(PDF)

S1 Table Frequency ratios of individual Tn mutants after exposure to 2.5 mM DEA/NO, 10 mM TBHP, or 0.25 mM H_2_O_2_. Tn mutants were removed from the analysis if they were not represented by at least 10 sequence reads in both untreated libraries. Zeroes in the treated samples were changed to 1 before calculating frequency (frequ.) ratios. Blank cells indicate that a particular Tn mutant was not detected or did not meet the inclusion criteria under a particular condition. Duplicate sequences (i.e. those that are present multiple times in the genome and thus cannot be mapped to a specific locus) are shaded in gray.
(XLSX)

S2 Table Overall frequency ratios for all genes after exposure to 2.5 mM DEA/NO, 10 mM TBHP, or 0.25 mM H_2_O_2_. Genes were removed from the analysis if they were not represented by at least 10 sequence reads in the untreated libraries. Zeroes in the treated samples were changed to 1 before calculating frequency (frequ.) ratios. Blank cells indicate that Tn mutants with insertions in a particular gene were not detected or did not meet the inclusion criteria under a particular condition. Genes are shaded in gray if any of the sequence reads from Tn mutants with insertions in the gene can be mapped to multiple sites in the genome.
(XLSX)

S3 Table Genes with an overall frequency ratio less than 0.5 in both replicates of at least one condition. Gray shading indicates a median frequency (frequ.) ratio <0.5. Genes were only included if the overall frequency ratio was <0.5 in both replicates of at least one condition. Blank boxes indicate that Tn mutants with insertions in that particular gene were not detected or did not meet the inclusion criteria under a particular condition.
(PDF)

S4 Table Genes with an overall frequency ratio greater than 2 in both replicates of at least one condition. Gray shading indicates a median frequency (frequ.) ratio >2. Genes were only included if the overall frequency ratio was >2 in both replicates of at least one condition. Genes were excluded if any of the sequences mapping within the gene could be mapped to multiple places on the chromosome. Blank boxes indicate that Tn mutants with insertions in that particular gene were not detected or did not meet the inclusion criteria under a particular condition.
(PDF)

S5 Table Transposon mutants used in this study. (PDF)

S6 Table Plasmids and other *B*. *burgdorferi* strains used in this study. (PDF)

S7 Table Sequences of primers used in this study. (PDF)
