# Supplementary material for: A high-throughput genetic screen identifies previously uncharacterized Borrelia burgdorferi genes important for resistance against reactive oxygen and nitrogen species
Source: PLoS Pathog. 2017 Feb 17;13(2):e1006225. doi: 10.1371/journal.ppat.1006225 (PMC5333916; doi:10.1371/journal.ppat.1006225)
Supplement: S1 Fig — The frequency of each mutant in the Tn library is shown for the two biological replicates of the untreated (A-C) and treated (D-F) samples. The nonparametric Spearman correlation coefficient R is shown in each case. Also shown are the nonparametric Spearman correlation coefficients comparing each of the 6 inocula used to infect 3 groups of C57BL/6 mice and 3 groups of gp91phox-/- mice with the mini-library of 39 Tn mutants (G). The frequency of each of these 39 Tn mutants in the three C57BL/6 and gp91phox-/- organ pools, along with the correlation coefficients between the samples, is also shown (H-I). (PDF) [file ppat.1006225.s001.pdf]

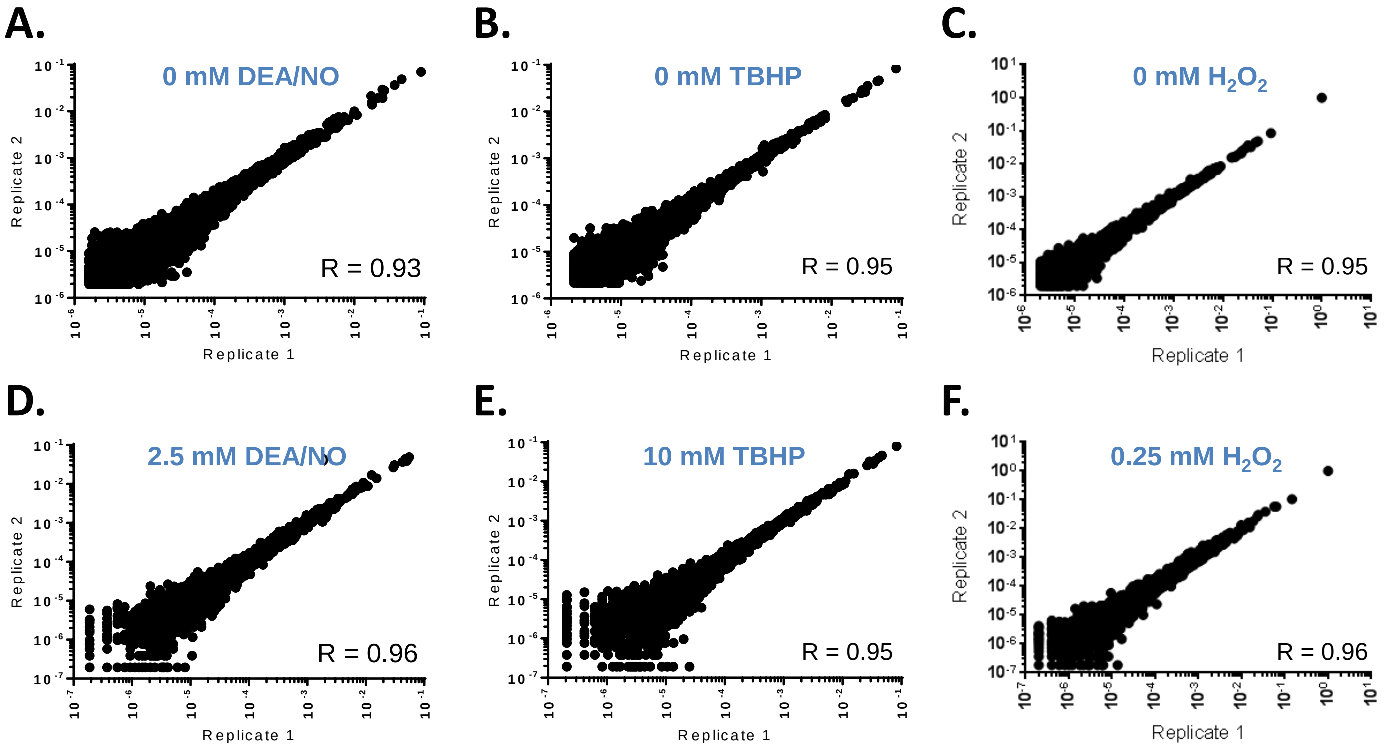

G.

	C57BL/6 Inocula 1	C57BL/6 Inocula 2	C57BL/6 Inocula 3	*gp91*$^{phox-/-}$ Inocula 1	*gp91*$^{phox-/-}$ Inocula 2	*gp91*$^{phox-/-}$ Inocula 3
C57BL/6 Inocula 1		0.73	0.72	0.53	0.52	0.52
C57BL/6 Inocula 2			0.99	0.90	0.89	0.89
C57BL/6 Inocula 3				0.90	0.89	0.90
gp91$^{phox-/}$ Inocula 1					0.99	0.99
gp91$^{phox-/}$ Inocula 2						0.99
gp91$^{phox-/}$ Inocula 3						

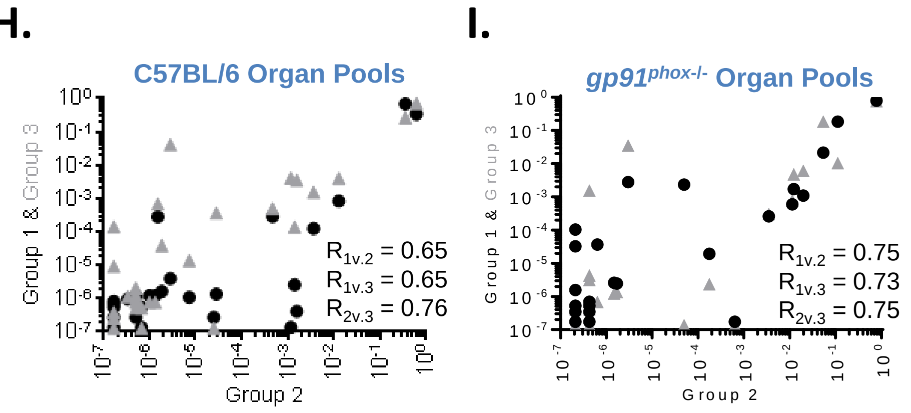

S1 Fig. Correlation between biological replicates of the Tn-seq experiments. The frequency of each mutant in the Tn library is shown for the two biological replicates of the untreated (A-C) and treated (D-F) samples. The nonparametric Spearman correlation coefficient R is shown in each case. Also shown are the nonparametric Spearman correlation coefficients comparing each of the 6 inocula used to infect 3 groups of C57BL/6 mice and 3 groups of *gp91*$^{phox-/-}$ mice with the mini-library of 39 Tn mutants (G). The frequency of each of these 39 Tn mutants in the three C57BL/6 and *gp91*$^{phox-/-}$ organ pools, along with the correlation coefficients between the samples, is also shown (H-I).
