# Supplementary material for: A high-throughput genetic screen identifies previously uncharacterized Borrelia burgdorferi genes important for resistance against reactive oxygen and nitrogen species
Source: PLoS Pathog. 2017 Feb 17;13(2):e1006225. doi: 10.1371/journal.ppat.1006225 (PMC5333916; doi:10.1371/journal.ppat.1006225)
Supplement: S2 Fig — The Tn::bb0431 and Tn::bb0839 Tn mutants were exposed to 1.25 mM DEA/NO, 1.25 mM diethylamine (DEA), or culture medium alone along with the parental strain 5A18NP1. Genomic equivalents were quantified following a three-day outgrowth period, and an outgrowth ratio was determined for each strain as the ratio of genomic equivalents in the treated sample compared to the untreated sample. *, P < 0.01 compared to 5A18NP1 by 2-way ANOVA followed by Dunnett’s test. N.S., not significant. (PDF) [file ppat.1006225.s002.pdf]

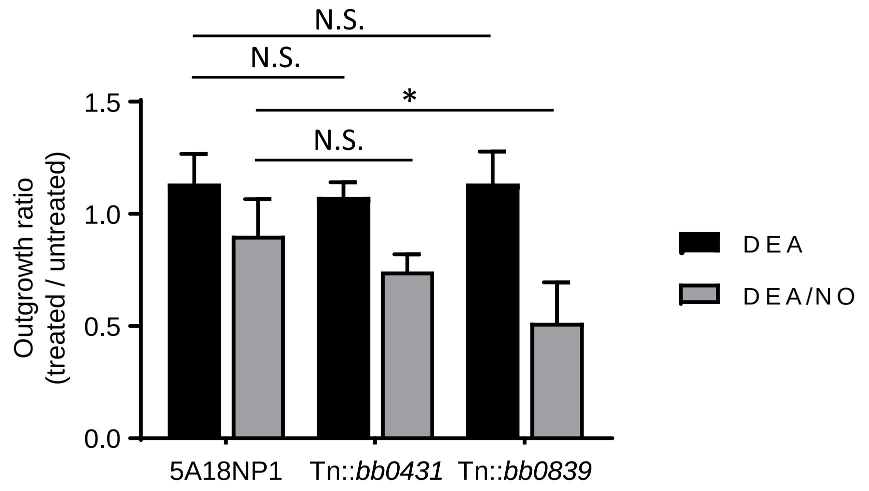

S2 Fig. Tn::*bb0431* and Tn::*bb0839* are not more sensitive to diethylamine compared to the parental strain. The Tn::*bb0431* and Tn::*bb0839* Tn mutants were exposed to 1.25 mM DEA/NO, 1.25 mM diethylamine (DEA), or culture medium alone along with the parental strain 5A18NP1. Genomic equivalents were quantified following a three-day outgrowth period, and an outgrowth ratio was determined for each strain as the ratio of genomic equivalents in the treated sample compared to the untreated sample. *, P < 0.01 compared to 5A18NP1 by 2-way ANOVA followed by Tukey's test. N.S., not significant.
