# Supplementary material for: A high-throughput genetic screen identifies previously uncharacterized Borrelia burgdorferi genes important for resistance against reactive oxygen and nitrogen species
Source: PLoS Pathog. 2017 Feb 17;13(2):e1006225. doi: 10.1371/journal.ppat.1006225 (PMC5333916; doi:10.1371/journal.ppat.1006225)
Supplement: S3 Fig — Total RNA was prepared from the parent 5A18NP1 (P), the Tn::bb0017 mutant (M), and the complemented strain DM104 (C), then subjected to RT-PCR using oligonucleotide primers specific for bb0017 (left half) and flaB (right half) as described in S7 Table. The absence or presence of reverse transcriptase (RT) in the samples is indicated by a minus or plus symbol, respectively. The leftmost lane contains a 100 base pair ladder with the 0.5 kb and 1 kb fragments indicated. (PDF) [file ppat.1006225.s003.pdf]

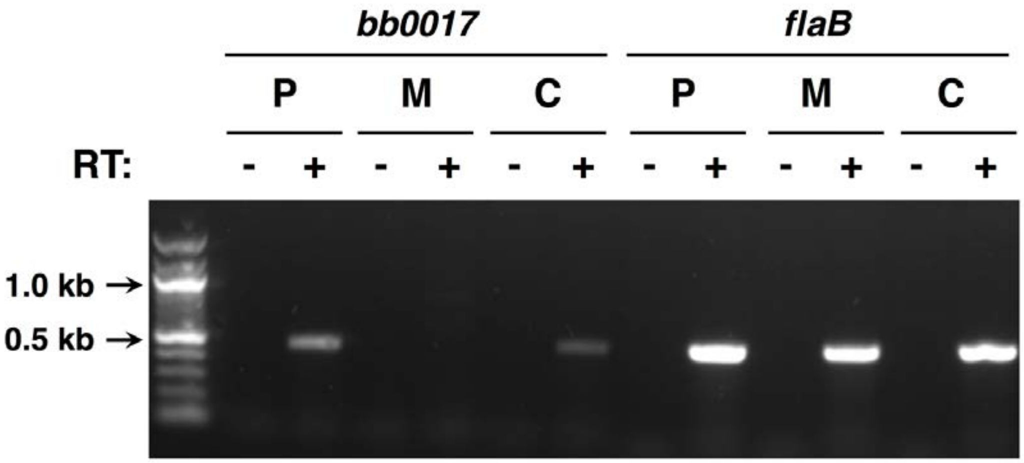


**S3 Fig. RT-PCR of the Tn::*bb0017* strain shows the loss of the *bb0017* transcript**. Total RNA was prepared from the parent 5A18NP1 (P), the Tn::*bb0017* mutant (M), and the complemented strain DM104 (C), then subjected to RT-PCR using oligonucleotide primers specific for *bb0017* (left half) and *flaB* (right half) as described in S7 Table. The absence or presence of reverse transcriptase (RT) in the samples is indicated by a minus or plus symbol, respectively. The leftmost lane contains a 100 base pair ladder with the 0.5 kb and 1 kb fragments indicated.
