# Supplementary material for: A high-throughput genetic screen identifies previously uncharacterized Borrelia burgdorferi genes important for resistance against reactive oxygen and nitrogen species
Source: PLoS Pathog. 2017 Feb 17;13(2):e1006225. doi: 10.1371/journal.ppat.1006225 (PMC5333916; doi:10.1371/journal.ppat.1006225)
Supplement: S4 Fig — Clustal Omega was used to align the amino acid sequence of BB1064 from B. burgdorferi (Bb_BB0164) with the amino acid sequences of homologs in Bacillus subtilis (Bs_YfkE), Methanococcus jannaschii (Mj_NCX), Saccharomyces cerevisiae (Sc_VCX1), and Arabidopsis thaliana (At_CAX1). Conserved amino acids are indicated by red shading or blue borders. Numbering corresponds to the BB0164 sequence. Green rectangles indicate the 11 transmembrane domains (TM0 –TM10) identified in the crystal structure of B. subtilis YfkE (Protein Data Bank ID code 4KJS). Orange rectangles indicate predicted alpha helices in BB0164 (Phyre2) [35]. Amino acids that are involved in Ca2+ transport in B. subtilis YfkE are indicated by asterisks [50]. This figure was prepared using ESPript [77]. (PDF) [file ppat.1006225.s004.pdf]

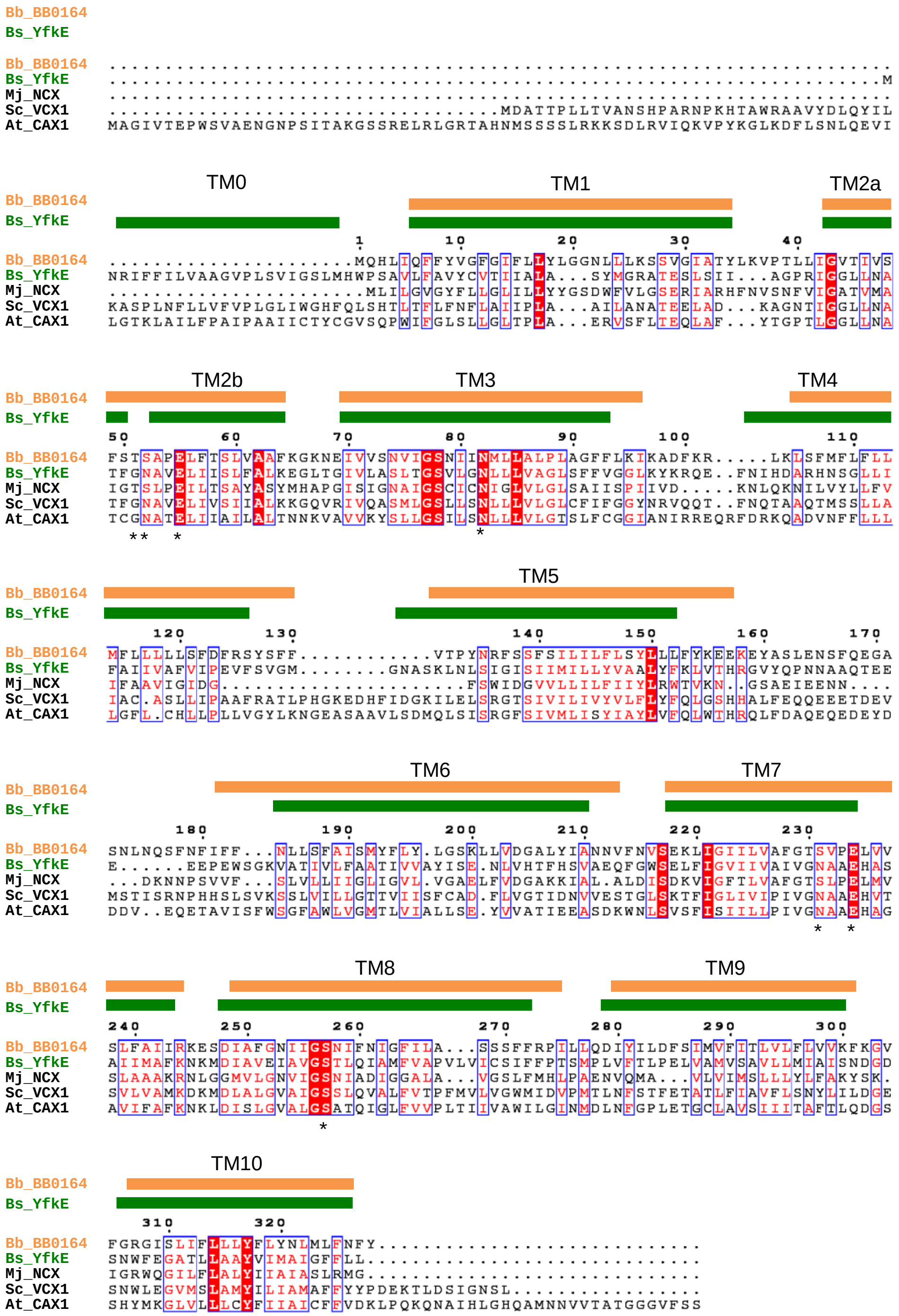

S4 Fig. Alignment of BB0164 with other CaCA transporter family members. ClustalW was used to align the amino acid sequence of BB1064 from *B. burgdorferi* (Bb_BB0164) with the amino acid sequences of homologs in *Bacillus subtilis* (Bs_YfkE), *Methanococcus jannaschii* (Mj_NCX), *Saccharomyces cerevisiae* (Sc_VCX1), and *Arabidopsis thaliana* (At_CAX1). Conserved amino acids are indicated by red shading or blue borders. Numbering corresponds to the BB0164 sequence. Green rectangles indicate the 11 transmembrane domains (TM0 – TM10) identified in the crystal structure of *B. subtilis* YfkE (Protein Data Bank ID code 4KJS). Orange rectangles indicate predicted alpha helices in BB0164 (Phyre^2)[a]. Amino acids that are involved in Ca^{2+} transport in *B. subtilis* YfkE are indicated by asterisks[b]. This figure was prepared using ESPript[c].

[a] [L.A. Kelley, S. Mezulis, C.M. Yates, M.N. Wass, and M.J. Sternberg. (2015) *Nat Protoc* **10**(6):845-58.]
[b] [M. Wu, S. Tong, S. Waltersperger, K. Diederichs, M. Wang, and L. Zheng. (2013) *PNAS* **110**(28):11367-72.]
[c] [X. Robert and P. Gouet. (2014) *Nucl Acids Res* **42**(W1):W320-W324.]
