# Supplementary material for: A high-throughput genetic screen identifies previously uncharacterized Borrelia burgdorferi genes important for resistance against reactive oxygen and nitrogen species
Source: PLoS Pathog. 2017 Feb 17;13(2):e1006225. doi: 10.1371/journal.ppat.1006225 (PMC5333916; doi:10.1371/journal.ppat.1006225)
Supplement: S3 Table — Gray shading indicates a median frequency (frequ.) ratio <0.5. Genes were only included if the overall frequency ratio was <0.5 in both replicates of at least one condition. Blank boxes indicate that Tn mutants with insertions in that particular gene were not detected or did not meet the inclusion criteria under a particular condition. (PDF) [file ppat.1006225.s007.pdf]

S3 Table. Genes with an overall frequency ratio less than 0.5 in both replicates of at least one stress condition. Gray shading indicates a median frequency (frequ.) ratio <0.5. Genes were only included if the overall frequency ratio was <0.5 in both replicates of at least one condition. Blank boxes indicate that Tn mutants with insertions in that particular gene were not detected or did not meet the inclusion criteria under a particular condition.

Rep	Locus	Annotation	**DEA/NO Frequ. Ratio** Median (Replicate 1, Replicate 2)		**TBHP Frequ. Ratio** Median (Replicate 1, Replicate 2)		**H_2O_2 Frequ. Ratio** Median (Replicate 1, Replicate 2)	
chr	*bb0025*	putative transcriptional regulator, YebC-like	0.230	(0.357, 0.103)			0.183	(0.320, 0.046)
chr	*bb0141*	BesA, membrane fusion protein	0.444	(0.414, 0.474)	2.188	(2.397, 1.980)	1.610	(1.949, 1.271)
chr	*bb0157*	hypothetical protein	0.408	(0.425, 0.391)	0.672	(0.718, 0.625)	0.774	(0.712, 0.836)
chr	*bb0223*	conserved hypothetical protein	0.417	(0.396, 0.438)	2.173	(2.330, 2.017)	2.259	(2.526, 1.992)
chr	*bb0224*	putative lipoprotein	0.417	(0.387, 0.448)	1.941	(2.094, 1.788)	1.722	(1.953, 1.490)
chr	*bb0254*	RecJ, single-stranded-DNA-specific exonuclease	0.476	(0.465, 0.488)	1.107	(1.063, 1.152)	1.096	(1.111, 1.082)
chr	*bb0267*	hypothetical protein	0.295	(0.299, 0.292)	1.047	(0.819, 1.275)	0.493	(0.642, 0.344)
chr	*bb0344*	UvrD, DNA helicase II	0.103	(0.108, 0.098)	1.295	(1.247, 1.343)	1.196	(1.125, 1.267)
chr	*bb0414*	CheR, chemotaxis protein	0.358	(0.377, 0.340)	0.876	(0.764, 0.988)	0.925	(1.301, 0.549)
chr	*bb0431*	CobQ/MinD nucleotide binding domain-containing protein	0.247	(0.293, 0.202)	1.880	(1.714, 2.045)	1.116	(1.433, 0.799)
chr	*bb0432*	conserved hypothetical protein	0.430	(0.420, 0.439)				
chr	*bb0439*	hypothetical protein	0.229	(0.420, 0.037)				
chr	*bb0457*	UvrC, excinuclease ABC subunit	0.048	(0.054, 0.041)	0.986	(1.262, 0.710)	0.984	(1.186, 0.781)
chr	*bb0467*	laccase domain-containing protein	0.162	(0.119, 0.205)				
chr	*bb0617*	hypothetical protein	0.294	(0.295, 0.292)	2.395	(3.782, 1.009)	0.791	(0.876, 0.706)
chr	*bb0618*	Cdd, cytidine deaminase	0.467	(0.484, 0.450)	0.862	(1.101, 0.622)	1.079	(0.950, 1.208)
chr	*bb0623*	Mfd, transcription-repair coupling factor	0.341	(0.311, 0.371)	1.202	(1.409, 0.995)	1.108	(1.254, 0.962)
chr	*bb0638*	Na^+/H^+ antiporter	0.449	(0.414, 0.484)	10.159	(10.799, 9.52)	0.284	(0.323, 0.245)
chr	*bb0749*	hypothetical protein	0.295	(0.385, 0.205)			1.124	(1.015, 1.234)
chr	*bb0758*	hypothetical protein	0.338	(0.439, 0.238)				
chr	*bb0803*	TruB, tRNA pseudouridine 55 synthase	0.376	(0.271, 0.481)	2.378	(2.001, 2.756)	0.164	(0.215, 0.113)
chr	*bb0825*	hypothetical protein	0.431	(0.381, 0.481)	1.240	(0.708, 1.772)	0.733	(0.655, 0.810)
chr	*bb0827*	HrpA, ATP-dependent helicase	0.303	(0.414, 0.192)				
chr	*bb0836*	UvrB, excinuclease ABC subunit	0.072	(0.110, 0.034)	0.650	(0.778, 0.523)	1.954	(2.012, 1.896)
chr	*bb0839*	putative ribonuclease HI	0.074	(0.049, 0.099)	0.739	(0.403, 1.075)	1.773	(1.155, 2.390)
lp54	*bba54*	hypothetical protein	0.243	(0.193, 0.293)	2.353	(3.976, 0.729)	0.924	(1.131, 0.718)
chr	*bb0017*	putative integral membrane protein	1.159	(1.138, 1.179)	0.231	(0.228, 0.233)	0.256	(0.265, 0.247)
chr	*bb0042*	PhoU, phosphate transport system regulatory protein	1.006	(0.773, 1.239)	0.414	(0.462, 0.366)	1.024	(1.624, 0.425)
chr	*bb0050*	putative energy coupling factor transporter	0.648	(0.624, 0.673)	0.398	(0.383, 0.412)	0.472	(0.483, 0.461)
chr	*bb0051*	putative energy coupling factor transporter	0.580	(0.458, 0.702)	0.385	(0.418, 0.351)	0.392	(0.400, 0.385)
chr	*bb0317*	riboflavin ABC transporter permease	0.892	(1.310, 0.473)	0.164	(0.169, 0.160)	0.660	(0.302, 1.018)
chr	*bb0319*	riboflavin ABC transporter solute-binding protein			0.401	(0.388, 0.413)	0.625	(1.015, 0.234)
chr	*bb0412*	hypothetical protein	0.976	(0.896, 1.056)	0.291	(0.288, 0.293)	0.249	(0.297, 0.201)
chr	*bb0420*	Hk1, sensory transduction histidine kinase	0.682	(0.724, 0.639)	0.371	(0.396, 0.346)	0.417	(0.449, 0.385)
chr	*bb0434*	Spo0J, stage 0 sporulation protein J	0.915	(0.064, 1.765)	0.110	(0.171, 0.050)	1.106	(1.575, 0.638)
chr	*bb0554*	carbon monoxide dehydrogenase subunit-like	1.103	(1.079, 1.127)	0.344	(0.331, 0.358)	0.312	(0.331, 0.293)
chr	*bb0555*	carbon monoxide dehydrogenase subunit-like	0.976	(1.005, 0.947)	0.314	(0.342, 0.287)	0.352	(0.391, 0.313)
chr	*bb0556*	carbon monoxide dehydrogenase subunit-like	0.921	(0.876, 0.965)	0.416	(0.471, 0.362)	0.274	(0.278, 0.271)
chr	*bb0631*	hypothetical protein	1.578	(0.912, 2.244)	0.257	(0.188, 0.326)	0.301	(0.304, 0.298)
cp26	*bbb16*	OppAIV, oligopeptide ABC transporter	2.033	(2.113, 1.954)	0.444	(0.451, 0.437)	0.783	(0.764, 0.802)
lp25	*bbe29*	pseudogene	1.216	(1.269, 1.162)	0.434	(0.436, 0.433)	0.168	(0.146, 0.190)
lp25	*bbe29a*	pseudogene	1.151	(1.223, 1.079)	0.446	(0.451, 0.441)	0.654	(0.658, 0.649)
chr	*bb0164*	putative K^+-dependent Na^+/Ca^+ exchanger-like protein	0.618	(0.457, 0.778)	0.603	(0.544, 0.661)	0.069	(0.085, 0.053)
chr	*bb0202*	putative CorC-like transporter protein, CBS domain	0.589	(0.558, 0.620)	0.677	(0.721, 0.633)	0.094	(0.101, 0.086)
chr	*bb0243*	GlpD, glycerol-3-phosphate dehydrogenase	0.610	(0.559, 0.661)	1.598	(1.631, 1.566)	0.205	(0.234, 0.176)
chr	*bb0347*	fibronectin binding protein	0.528	(0.457, 0.600)	0.733	(0.804, 0.661)	0.263	(0.300, 0.226)
chr	*bb0363*	PdeA, cyclic-di-GMP phosphodiesterase	0.637	(0.581, 0.692)	1.330	(1.407, 1.254)	0.224	(0.234, 0.215)
chr	*bb0411*	putative DNA/RNA non-specific endonuclease	0.930	(0.881, 0.979)	0.513	(0.562, 0.463)	0.313	(0.337, 0.290)
chr	*bb0473*	hypothetical protein	0.288	(0.063, 0.513)			0.077	(0.056, 0.097)
chr	*bb0530*	conserved hypothetical protein	0.701	(0.741, 0.660)	0.824	(0.881, 0.767)	0.419	(0.476, 0.361)
chr	*bb0597*	methyl-accepting chemotaxis protein	0.563	(0.359, 0.767)	0.720	(0.855, 0.584)	0.438	(0.414, 0.462)
chr	*bb0619*	DhhP, cyclic-di-AMP phosphodiesterase	0.657	(0.721, 0.592)	1.128	(1.250, 1.005)	0.402	(0.349, 0.455)
chr	*bb0669*	CheA2, chemotaxis protein					0.114	(0.033, 0.195)
chr	*bb0723*	CyaB, adenylyl cyclase	0.876	(0.603, 1.149)	0.681	(0.536, 0.826)	0.429	(0.424, 0.433)
chr	*bb0797*	MutS, DNA mismatch repair protein	0.619	(0.579, 0.659)	0.841	(0.859, 0.822)	0.335	(0.382, 0.288)
chr	*bb0823*	hypothetical protein	0.663	(0.447, 0.879)	1.142	(0.845, 1.439)	0.254	(0.118, 0.390)

chr	*bb0829*	exonuclease SbcD	0.599	(0.605, 0.593)	0.838	(0.948, 0.727)	0.265	(0.260, 0.269)
lp54	*bba14*	borrelia orf-D family	0.905	(1.135, 0.676)	0.729	(0.835, 0.623)	0.184	(0.169, 0.198)
lp54	*bba42*	hypothetical protein	0.699	(0.802, 0.596)	0.688	(0.727, 0.648)	0.263	(0.195, 0.331)
lp54	*bba43*	hypothetical protein	0.646	(0.663, 0.629)	0.638	(0.613, 0.663)	0.315	(0.396, 0.233)
cp26	*bbb06*	ChbB, chitobiose transporter protein	1.111	(1.003, 1.219)	0.485	(0.519, 0.451)	0.101	(0.093, 0.108)
lp28-1	*bbf14*	hypothetical protein	1.973	(0.964, 2.982)			0.424	(0.406, 0.443)
lp28-2	*bbg02*	hypothetical protein	1.049	(0.973, 1.125)	0.771	(0.869, 0.672)	0.124	(0.127, 0.122)
lp36	*bbk48*	immunogenic protein P37	1.136	(1.141, 1.130)	0.800	(0.764, 0.836)	0.262	(0.257, 0.267)
lp36	*bbk52*	protein p23	1.112	(1.197, 1.027)	0.783	(0.791, 0.775)	0.235	(0.210, 0.260)
cp9	*bbc07*	hypothetical protein	1.007	(0.997, 1.018)	0.720	(0.686, 0.755)	0.122	(0.109, 0.134)

Rep, replicon
Chr, chromosome
