# Supplementary material for: A high-throughput genetic screen identifies previously uncharacterized Borrelia burgdorferi genes important for resistance against reactive oxygen and nitrogen species
Source: PLoS Pathog. 2017 Feb 17;13(2):e1006225. doi: 10.1371/journal.ppat.1006225 (PMC5333916; doi:10.1371/journal.ppat.1006225)
Supplement: S4 Table — Gray shading indicates a median frequency (frequ.) ratio >2. Genes were only included if the overall frequency ratio was >2 in both replicates of at least one condition. Genes were excluded if any of the sequences mapping within the gene could be mapped to multiple places on the chromosome. Blank boxes indicate that Tn mutants with insertions in that particular gene were not detected or did not meet the inclusion criteria under a particular condition. (PDF) [file ppat.1006225.s008.pdf]

**S4 Table. Genes with an overall frequency ratio greater than 2 in both replicates of at least one condition.** Gray shading indicates a median frequency (frequ.) ratio >2. Genes were only included if the overall frequency ratio was >2 in both replicates of at least one condition. Genes were excluded if any of the sequences mapping within the gene could be mapped to multiple places on the chromosome. Blank boxes indicate that Tn mutants with insertions in that particular gene were not detected or did not meet the inclusion criteria under a particular condition.

| Rep | Locus | Annotation | **DEA/NO Frequ. Ratio** Median (Replicate 1, Replicate 2) | **TBHP Frequ. Ratio** Median (Replicate 1, Replicate 2) | **$H_2O_2$ Frequ. Ratio** Median (Replicate 1, Replicate 2) |
|---|---|---|---|---|---|
| chr | *bb0073* | hypothetical protein | 2.442 (1.919, 2.965) | 3.150 (2.352, 3.949) | 1.723 (2.791, 0.656) |
| chr | *bb0001* | hypothetical protein | 0.533 (0.543, 0.523) | 2.369 (2.627, 2.111) | 1.698 (1.707, 1.688) |
| chr | *bb0165* | hypothetical protein | 0.610 (0.702, 0.517) | 2.573 (2.628, 2.519) | 0.950 (1.159, 0.741) |
| chr | *bb0223* | conserved hypothetical protein | 0.417 (0.396, 0.438) | 2.173 (2.33, 2.017) | 2.259 (2.526, 1.992) |
| chr | *bb0368* | GpsA, glycerol-3-phosphate dehydrogenase | 0.828 (1.165, 0.49) | 5.895 (6.76, 5.031) | 0.558 (0.515, 0.602) |
| chr | *bb0516* | RNA methyltransferase, TrmH family | 0.850 (0.505, 1.196) | 3.605 (3.814, 3.395) | 0.632 (0.71, 0.553) |
| chr | *bb0525* | hypothetical protein | 0.508 (0.399, 0.617) | 3.297 (3.666, 2.928) | 1.269 (1.43, 1.108) |
| chr | *bb0637* | $Na^+/H^+$ antiporter | 0.576 (0.537, 0.615) | 7.339 (8.056, 6.622) | 1.134 (1.475, 0.792) |
| chr | *bb0638* | $Na^+/H^+$ antiporter | 0.449 (0.414, 0.484) | 10.159 (10.799, 9.52) | 0.284 (0.323, 0.245) |
| chr | *bb0803* | TruB, tRNA pseudouridine 55 synthase | 0.376 (0.271, 0.481) | 2.378 (2.001, 2.756) | 0.164 (0.215, 0.113) |
| lp28-2 | *bbg30* | hypothetical protein | 1.994 (1.783, 2.205) | 2.887 (2.31, 3.465) | 3.698 (2.04, 5.356) |
| cp32-4 | *bbr32* | hypothetical protein | 1.699 (1.537, 1.861) | 2.301 (2.498, 2.104) | 1.736 (1.521, 1.952) |
| chr | *bb0671* | CheX, chemotaxis protein | 0.789 (0.719, 0.859) | 1.678 (1.718, 1.639) | 2.302 (2.467, 2.137) |
| chr | *bb0831* | XylR-2, xylose operon regulatory protein | 0.781 (0.707, 0.855) | 1.832 (1.952, 1.713) | 2.730 (2.957, 2.502) |
| chr | *bb0840* | lipoprotein | 0.428 (0.281, 0.575) | 1.077 (1.028, 1.127) | 2.560 (2.622, 2.498) |
| lp28-1 | *bbf03* | RepU | 1.060 (1.121, 0.998) | 0.755 (0.668, 0.842) | 2.805 (2.626, 2.983) |
| lp28-2 | *bbg33* | hypothetical protein | | 0.996 (1.253, 0.739) | 4.924 (5.038, 4.81) |
| lp28-3 | *bbh0042* | hypothetical protein | 1.257 (1.368, 1.146) | 1.307 (1.485, 1.128) | 4.051 (3.673, 4.429) |
| lp38 | *bbj09* | OspD, outer surface protein D | 0.966 (0.868, 1.064) | 1.305 (1.212, 1.397) | 2.131 (2.235, 2.026) |
| lp38 | *bbj13* | hypothetical protein | 1.157 (1.063, 1.25) | 1.356 (1.56, 1.151) | 2.201 (2.312, 2.091) |
| lp36 | *bbk13* | hypothetical protein | 1.086 (1.081, 1.091) | 1.428 (1.499, 1.358) | 2.184 (2.098, 2.27) |

Rep, replicon

Chr, chromosome
