# Supplementary material for: A high-throughput genetic screen identifies previously uncharacterized Borrelia burgdorferi genes important for resistance against reactive oxygen and nitrogen species
Source: PLoS Pathog. 2017 Feb 17;13(2):e1006225. doi: 10.1371/journal.ppat.1006225 (PMC5333916; doi:10.1371/journal.ppat.1006225)
Supplement: S5 Table — (PDF) [file ppat.1006225.s009.pdf]

S5 Table. Transposon mutants used in this study.

Strain Name[a]	Tn mutant clone name[b]	Gene	Tn insertion site (Ratio)[c]	Tn orientation / Gene orientation	Missing plasmids[d]
Tn::*bb0017*-1	T05TC306		16662 (0.85)	Reverse / Forward	lp5
Tn::*bb0017*-2	T09TC420		16263 (0.44)	Forward / Forward	lp28-1
Tn::*bb0025*-1	T11TC001		24097 (0.82)	Forward / Reverse	lp21, lp5
Tn::*bb0050*	T09TC314		47349 (0.27)	Reverse / Forward	cp32-6, cp9
Tn::*bb0141*-1	T08TC099	*besA*	142557 (0.39)	Reverse / Reverse	cp9, lp5
Tn::*bb0141*-2	T08TC113	*besA*	142800 (0.14)	Reverse / Reverse	--
Tn::*bb0157*	T05TC360		158215 (0.38)	Reverse / Forward	lp5
Tn::*bb0164*	T11P02D04		164997 (0.91)	Reverse / Reverse	lp5
Tn::*bb0202*	T06TC525		203539 (0.56)	Reverse / Forward	lp5
Tn::*bb0317*	T05TC132		323719 (0.07)	Reverse / Reverse	lp5
Tn::*bb0319*	T06TC392		325858 (0.41)	Forward / Reverse	--
Tn::*bb0328*-B	T03TC095	*oppA1*	335334 (0.26)	Reverse / Forward	--
Tn::*bb0328*-D	T06TC172	*oppA1*	335793 (0.55)	Forward / Forward	--
Tn::*bb0328*-F	T04TC011	*oppA1*	336241 (0.83)	Reverse / Forward	cp9
Tn::*bb0344*-1	T07TC421	*uvrD*	351622 (0.87)	Reverse / Reverse	lp36, lp5
Tn::*bb0344*-2	T10TC209	*uvrD*	353286 (0.07)	Forward / Reverse	--
Tn::*bb0347*	T03TC130		356639 (0.81)	Forward / Forward	lp5
Tn::*bb0363*	T05TC151	*pdeA*	371285 (0.13)	Forward / Forward	lp5
Tn::*bb0368*	T05TC189	*gpsA*	376509 (0.50)	Reverse / Reverse	--
Tn::*bb0411*	T09TC436	*nucA*	422727 (0.44)	Reverse / Forward	cp32-1
Tn::*bb0412*	T05TC588		423620 (0.73)	Forward / Forward	lp5
Tn::*bb0414*-1	T11TC527	*cheR2*	425058 (0.42)	Forward / Forward	lp5
Tn::*bb0414*-2	T08TC139	*cheR2*	425339 (0.75)	Reverse / Forward	--
Tn::*bb0431*-1	T06TC141		449738 (0.43)	Reverse / Forward	lp38, lp5
Tn::*bb0457*-1	T05TC116	*uvrC*	477018 (0.58)	Reverse / Reverse	lp28-2
Tn::*bb0457*-2	T10TC161	*uvrC*	477982 (0.05)	Forward / Reverse	lp21, lp5, cp9
Tn::*bb0473*	T04TC218		493861 (0.93)	Reverse / Forward	cp9
Tn::*bb0554*	T09TC312		566269 (0.17)	Forward / Forward	lp5
Tn::*bb0555*	T05TC323		568021 (0.45)	Reverse / Forward	lp5
Tn::*bb0556*	T10TC010		568449 (0.21)	Forward / Forward	cp9, lp5
Tn::*bb0637*	T06TC391	*nhaC1*	676105 (0.80)	Forward / Forward	lp21
Tn::*bb0638*-1	T03TC062	*nhaC2*	676848 (0.27)	Reverse / Forward	--
Tn::*bb0638*-2	T11TC428	*nhaC2*	677586 (0.80)	Forward / Forward	lp5
Tn::*bb0723*	T11TC373	*cyaB*	762143 (0.02)	Forward / Reverse	cp32-4
Tn::*bb0803*-1	T08TC365	*truB*	848006 (0.61)	Reverse / Forward	--
Tn::*bb0831*	T11TC542	*xylR2*	879785 (0.51)	Reverse / Forward	--
Tn::*bb0839*-1	T09TC031		897146 (0.92)	Forward / Reverse	--
Tn::*bb0839*-2	T09TC009		897244 (0.77)	Reverse / Reverse	--
Tn::*bbb06*	T04TC080	*chbB*	4594 (0.49)	Forward / Forward	--
Tn::*bbe22*	T01P01A11[e]	*pncA*	15343 (0.56)	Reverse / Reverse	lp5

Tn, transposon

[a] All Tn mutants listed in this table (with the exception of Tn::*bb0328*-D) were pooled to generate the mini-library of 39 Tn mutants used for the *in vivo* Tn-seq experiment

[b] Clone names as described [T. Lin, L. Gao, C. Zhang, E. Odeh, M.B. Jacobs, L. Coutte, G. Chaconas, M.T. Philipp, and S.J. Norris, PLoS One 7(10):e47532]

[c] Ratio = Tn insertion site / 3' position of gene

[d] In addition to lp56 and lp28-4, which are absent in 5A18NP1, the parental strain of the Tn library

[e] T01P01A11 also contains a second Tn insertion in *hk1*.
