# Supplementary material for: A high-throughput genetic screen identifies previously uncharacterized Borrelia burgdorferi genes important for resistance against reactive oxygen and nitrogen species
Source: PLoS Pathog. 2017 Feb 17;13(2):e1006225. doi: 10.1371/journal.ppat.1006225 (PMC5333916; doi:10.1371/journal.ppat.1006225)
Supplement: S6 Table — (PDF) [file ppat.1006225.s010.pdf]

S6 Table. Plasmids and other *B. burgdorferi* strains used in this study

Plasmid / Strain name	Description[a]	Resistance[b]	Reference / Source
**Plasmids**			
pCR-Blunt	PCR cloning vector	kan^R	Thermo-Fisher Scientific
pJH508	*bb0017 cis* complement allelic exchange vector; used to generate strain DM104	kan^R, $strep^R$	This study
pJH511	P_{bb0165}-*bb0164 trans* complement allelic exchange vector at a remote site in the chromosome; used to generate strain JH511	kan^R, $strep^R$	This study
***B. burgdorferi* strains**			
297	*B. burgdorferi* strain, parental strain of OY04		(Ouyang et al., 2009)[c]
OY04	*B. burgdorferi bmtA* mutant	kan^R	(Ouyang et al., 2009)[c]
5A18NP1	*B. burgdorferi* B31 clone, parental strain of Tn library ($lp56^-$, $lp28\text{-}4^-$)	kan^R	(Kawabata et al., 2004)[d]
DM104	Tn::*bb0017*-comp; *cis* complementation of *bb0017* under native promoter in T05TC306 ($lp56^-$, $lp28\text{-}4^-$, $lp5^-$)	kan^R, $strep^R$	This study
JH511	Tn::*bb0164*-comp; *trans* complementation of *bb0164* under native promoter at the *bb0445-bb0446* locus in T11P02D04 ($lp56^-$, $lp28\text{-}4^-$, $lp21^-$, $lp5^-$)	kan^R, $gent^R$, $strep^R$	This study

[a] Missing plasmids are indicated if applicable
[b] kan^R, kanamycin resistant; $strep^R$, streptomycin resistant; $gent^R$, gentamicin resistant
[c] [Z. Ouyang, M. He, T. Oman, X.F. Yang, and M.V. Norgard. (2009) *PNAS* **106**(9):3449-3454.]
[d] [H. Kawabata, S.J. Norris, and H. Watanabe. (2004) *Infect Immun* **72**(12):7147-54.]
