# Supplementary material for: A high-throughput genetic screen identifies previously uncharacterized Borrelia burgdorferi genes important for resistance against reactive oxygen and nitrogen species
Source: PLoS Pathog. 2017 Feb 17;13(2):e1006225. doi: 10.1371/journal.ppat.1006225 (PMC5333916; doi:10.1371/journal.ppat.1006225)
Supplement: S7 Table — (PDF) [file ppat.1006225.s011.pdf]

S7 Table. Sequences of primers used in this study

**Primer Name**	**Description**	**Sequence** [a]
pJH508 F1F	*bb0017* & upstream region	CGTAAAGGGGCATATCTATTGGAC
pJH508 F1R	*bb0017* & upstream region	CTTCCTTGAAGCTCGGGTATTTAAATTTCTTGTTTTAATGAG
pJH508 F2F	P$_{flgB}$-*aadA* for *bb0017* complement	CTCATTAAAACAAGAAATTTAAATACCCGAGCTTCAAGGAAG
pJH508 F2R	P$_{flgB}$-*aadA* for *bb0017* complement	GGTTCTTTTTTTAAGAGATTTTTAATTTATTTGCCGACTACCTTGGTG
pJH508 F3F	downstream region of *bb0017*	CACCAAGGTAGTCGGCAAATAAATTAAAAATCTCTTAAAAAAAGAACC
pJH508 F3R	downstream region of *bb0017*	GAGATTGTTGATGAGATAACTGAGAC
pJH511 F1F	*bb0445* region for allelic exchange	TATGCCTATGCAAAAAGCAGATG
pJH511 F1R	*bb0445* region for *bb0164* complement	GAGGAAGATTTTGATTGCTTGGTTAACATCGATCAAAAAGCAGCTTGC
pJH511 F2F	P$_{bb0165}$	GCAAGCTGCTTTTTGATCGATGTTAACCAAGCAATCAAAATCTTCCTC
pJH511 F2R	P$_{bb0165}$	CTCCTTTACAAGAAACAACAAATACATTAATACATGATACTTGC
pJH511 F3F	*bb0164*	GCAAGTATCATGTATTAATGTATTTGTTGTTTCTTGTAAAGGAG
pJH511 F3R	*bb0164*	CTTCCTTGAAGCTCGGGTATCAATAAAAGTTAAATAAC
pJH511 F4F	P$_{flgB}$-*aadA* for *bb0164* complement	GTTATTTAACTTTTATTGATACCCGAGCTTCAAGGAAG
pJH511 F4R	P$_{flgB}$-*aadA* for *bb0164* complement	CCATAATGCAAGCTCTGCCATATTATTTGCCGACTACCTTGGTG
pJH511 F5F	*bb0446* region for *bb0164* complement	ACCAAGGTAGTCGGCAAATAATATGGCAGAGCTTGCATTATGG
pJH511 F5R	*bb0446* region for allelic exchange	GCAAGTGAAAACTCAAAACTTGAATAC
bb0017 F	RT-PCR of *bb0017*	GGATCTTCAGGGGGATCAG
bb0017 R	RT-PCR of *bb0017*	TTAAATTTCTTGTTTTAATGAGATTGG
flaB F	RT-PCR of *flaB*	GGGAACTTGATTAGCCTGCGC
flaB R	RT-PCR of *flaB*	GGGTCTCAAGCGTCTTGGAC
bb0164 qPCR F2	RT-qPCR for *bb0164* (upstream of Tn insertion)	AACATTATTGGTAGTAATATTTTTAATATC
bb0164 qPCR R	RT-qPCR for *bb0164* (downstream of Tn insertion)	CAAAAACCCCTTTAAATTTAACC
bmtA qPCR F	RT-qPCR for *bmtA*	AAACCGCATAAGCACCCATAAGCC
bmtA qPCR R	RT-qPCR for *bmtA*	TTTGGAGCCTTGGCGTCTAATCCA
flaB qPCR F	RT-qPCR for *flaB*	GCAGCTAATGTTGCAAATCTTTTC
flaB qPCR R	RT-qPCR for *flaB*	GCAGGTGCTGGCTGTTGA

a Underline denotes overlap region engineered into primer design.
